# Supplementary material for: Can prediction error explain predictability effects on the N1 during picture-word verification?
Source: Imaging Neurosci (Camb). 2024 Apr 8;2:imag-2-00131. doi: 10.1162/imag_a_00131 (PMC12247556; doi:10.1162/imag_a_00131)
Supplement: Supplementary Material [file imag_a_00131-supp.pdf]

Supplementary Materials: Can Prediction Error Explain Predictability Effects on the N1 during Picture-Word Verification?

Contents

A Picture-Word Stimuli **3**

B Behavioural Validation Results **18**

C Localiser Task Stimuli **22**

D Statistical Power Analysis **30**

E Instructions given to Participants **37**

F Change to the High-Pass Filter Cut-Off **39**

Maximal Electrode Analysis 39

Full ERP Analysis 40

G Behavioural Results from the Picture-Word Task **43**

Response Times Results 43

Accuracies Results 45

H Results from the Localiser Task **50**

ERP Results 50

Behavioural Results 51

I Exploratory Alterations to the Maximal Electrode Method **56**

Using the Word-Noise Difference 56

Using Region-of-Interest Averages 57

J Model Estimates for the N400 **60**

K Checking ERPs Time-Locked to Pictures **61**

L Checking ERPs Time-Locked to Responses **64**

A Picture-Word Stimuli

There were 200 words per Congruency condition, with one congruent and one incongruent word per image. The experimental stimuli are summarised in **Figure 1**. First, stimuli were subset according to norms collected by Brysbaert et al. (2019), such that at least 90% of participants knew each word. Stimuli were additionally subset such that all words were nouns according to the dominant part of speech data from SUBTLEX-UK (van Heuven et al., 2014), and had a mean concreteness rating above 4 (on a Likert scale from 1, least concrete, to 5, most concrete) according to Brysbaert et al. (2014). Images were taken from the Bank of Online Standardised Stimuli (BOSS) norms (Brodeur et al., 2014), a large database of images with normed statistics, including percentage of name agreement, which, critically, we used as a measure of Predictability. Words were identified as possible picture-congruent words if they were listed as the most frequent (i.e., modal) name for any image in the BOSS norms, and were identified as possible picture-incongruent words if they were not.

Picture-congruent and -incongruent words were matched item-wise across five lexical variables, with specific tolerance ranges, as follows: (1) word length (number of characters), exactly; (2) concreteness according to Brysbaert et al. (2014), within $\pm.25$; (3) Zipf frequency (a logarithmic scale of word frequency) according to SUBTLEX-UK, within $\pm.125$; (4) character bigram probability (calculated from SUBTLEX-UK), within $\pm.0025$; and (5) OLD20 (the average Orthographic Levenshtein Distance of the 20 closest neighbours to a given word; Yarkoni et al., 2008) calculated from the LexOPS inbuilt dataset, within $\pm.75$. To ensure that picture-incongruent words were not inadvertent possible descriptors for images, the cosine positive pointwise mutual information (PPMI) measure of associative semantic similarity calculated from the Small World of Words (SWOW) word association norms (De Deyne et al., 2019) was minimised to be $\leq.01$ between each image's matched picture-congruent and picture-incongruent words. To ensure picture-incongruent words did not share orthographic features with their respective

Figure 1

Summary of the picture-word stimuli.

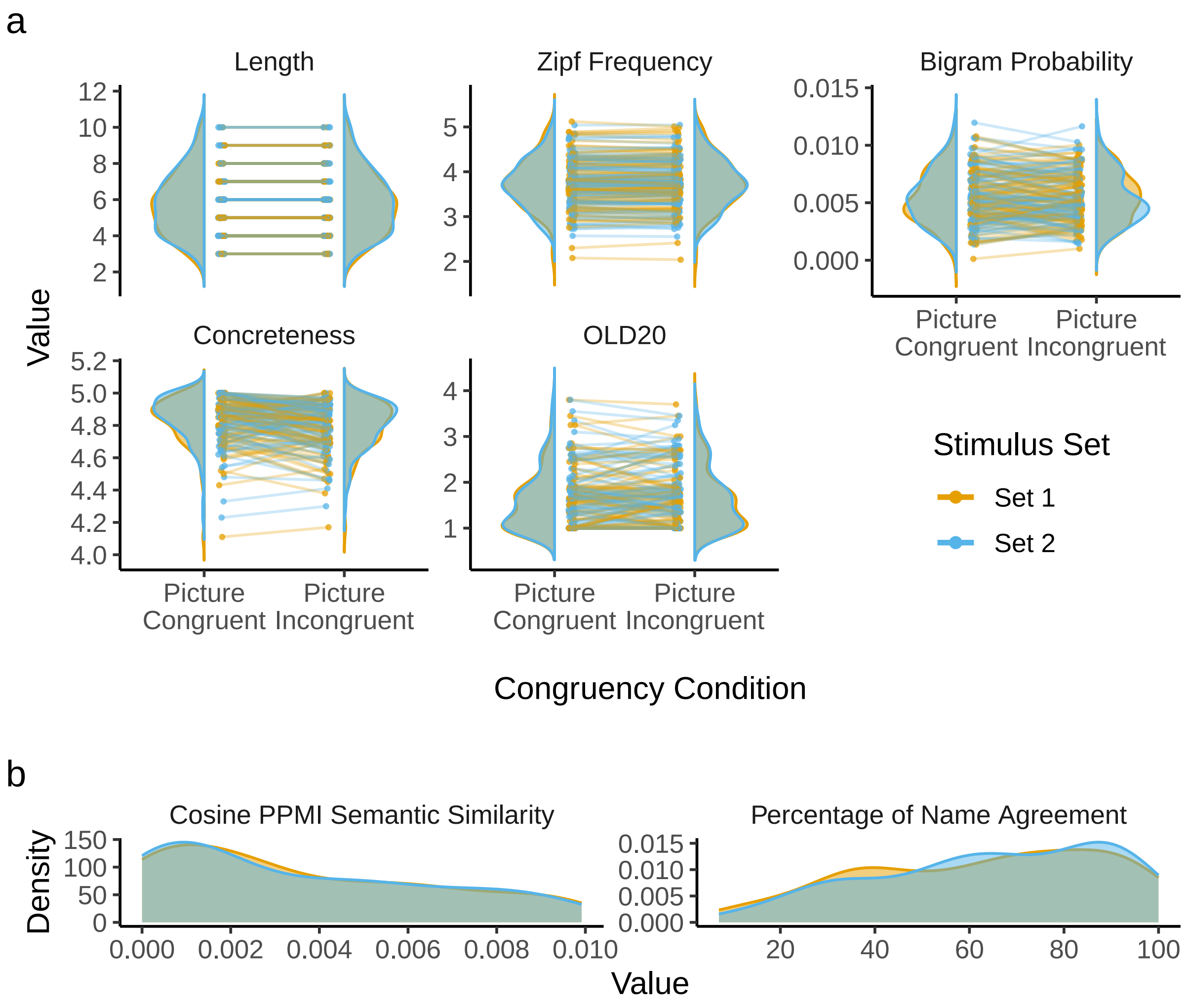

Each panel depicts how a single variable was controlled. (**a**) Probability densities for variables which were matched item-wise between picture-congruent and picture-incongruent conditions, and distribution-wise between counterbalanced stimulus Sets 1 (in *yellow*) and 2 (in *blue*). Points representing pairs of words which are matched item-wise are joined by lines. Points' positions are jittered slightly along the x-axis for visibility. (**b**) Probability densities for two variables matched only in a distribution-wise manner between the counterbalanced stimulus sets: Cosine PPMI (Positive Pointwise Mutual Information) Semantic Similarity from SWOW (Small World of Words; De Deyne et al., 2019), and modal name agreement from the BOSS norms. These variables cannot be matched between Congruency conditions because only a single value describes each matched congruent-incongruent word pair.

picture-congruent words, orthographic Levenshtein distance between matched items was maximised. As items were also matched in word length, this meant all matched pairs of

words had a Levenshtein distance equal to their number of characters. The variable used to index the Predictability of picture-congruent words was percentage of modal name agreement, which was sampled pseudo-randomly (picture-congruent words were not selected if no incongruent match could be identified fitting the constraints specified above) from the BOSS norms, and varied continuously in the generated stimuli from 7 to 100%.

As the participants were recruited in the United Kingdom, possible congruent and incongruent picture-word pairs were excluded if we identified the words as less frequent in British English (e.g., *sidewalk*) or if they were modal names for images that the Canadian participants of the BOSS norms are likely to have been more able to name or distinguish (e.g., *buffalo*, *bison*). In addition, picture-word pairs were excluded if words were identified as shortened versions of nouns (e.g., *limo*, *chimp*) or alternate names for the same object (e.g., *motorbike*, *motorcycle*). Candidate picture-incongruent words were additionally excluded if images were not representative of the images in the BOSS (e.g., *waiter* or *church*, as there were no other images of people or entire buildings in the BOSS), or if they were unimageable despite their high concreteness value (e.g., *item*). Plural words (e.g., *sticks*) were excluded, as most images in the BOSS have modal names that are singular. Finally, four images with modal names *nut*, *trumpet*, *spinach*, and *tuba* were excluded, as we judged these names to be incorrect descriptions of their images.

To avoid repetition effects, each image was presented once, with participants viewing either the associated picture-congruent or picture-incongruent word. This was counterbalanced by splitting the stimuli pseudo-randomly into two equally sized stimulus sets, referred to as Set 1 and Set 2. Each participant was presented with only one of these stimulus sets. Pictures followed by congruent words in Set 1 were followed by incongruent words in Set 2, and vice versa. To minimise any systematic difference between the counterbalanced groups, the split of stimuli was selected to maximise the empirical distributional overlap (Pastore & Calcagnì, 2019) between the two stimulus sets in relevant variables. Specifically, the stimulus sets were selected from 50,000 random splits to maximise the overlap between the

distributions of the following seven variables: (1) percentage of modal name agreement according to the BOSS norms; (2) cosine PPMI semantic similarity according to the SWOW; (3, 4) Zipf word frequency and character bigram probability according to SUBTLEX-UK; (5) word concreteness (Brysbaert et al., 2014); (6) word length; and (7) OLD20. Variables that were also matched item-wise between the conditions were matched distribution-wise separately within each Congruency condition. This ensured there were minimal systematic differences in distributions between conditions or stimulus sets.

To generate stimuli for practice trials, 20 matched pairs of picture-congruent and -incongruent words were generated using the same pipeline as above, except that word frequency, word concreteness, and character bigram probability were not matched item-wise. The practice stimuli were generated from images and words not used in the experimental stimuli. The same practice trials were presented to all participants.

Table 1

All stimuli for the picture-word task. Column Image IDs are unique file names given to each image in the BOSS, while %Agree reports the percentage of modal name agreement for the image in the BOSS. Set refers to the assigned stimulus sets. Column Word *contains the matched congruent* (C) *and incongruent* (I) *words associated with each image. The remaining columns are as follows, separating values into those for the congruent* (C) *and incongruent* (I) *words where possible: Length = number of characters;* Zipf *= Zipf frequency in SUBTLEX-UK;* OLD20 *= OLD20 values in the LexOPS dataset;* BG *= mean character bigram probabilities in SUBTLEX-UK;* CNC *= mean concreteness ratings in Brysbaert et al. (2014);* Cosine PPMI *= cosine positive pointwise mutual information values of semantic associative similarity between matched congruent and incongruent words from the Small World of Words. Rows are numbered for ease of reference.*

	Image ID	%Agree	Set	Word		Length		Zipf		OLD20		BG		CNC		Cosine PPMI
				C	I	C	I	C	I	C	I	C	I	C	I	
1	joustingspear	7%	2	spear	porch	5	5	3.42	3.47	1.20	1.15	.0062	.0047	5.00	4.92	.0071
2	cabasa	10%	1	shaker	trough	6	6	3.35	3.32	1.00	1.65	.0092	.0067	4.11	4.17	.0073
3	powerchair	10%	1	scooter	missile	7	7	3.63	3.66	1.60	1.70	.0077	.0057	4.96	4.83	.0065
4	pottery	12%	1	pottery	rainbow	7	7	4.14	4.18	1.65	2.40	.0070	.0069	4.72	4.57	.0093
5	lbracket01	13%	1	bracket	tornado	7	7	3.40	3.49	1.75	2.10	.0036	.0059	4.43	4.53	.0003
6	flail	14%	2	mace	knob	4	4	3.37	3.50	1.00	1.35	.0042	.0029	4.81	4.75	.0017
7	plastictube	16%	1	tube	chip	4	4	4.28	4.26	1.00	1.00	.0031	.0051	4.82	4.71	.0008
8	paintscraper	16%	2	scraper	nightie	7	7	2.80	2.89	1.75	1.85	.0052	.0037	4.23	4.30	.0002
9	pillar	19%	2	pillar	sewage	6	6	3.54	3.58	1.60	1.95	.0060	.0041	4.77	4.52	.0016
10	bazooka	19%	1	bazooka	sunburn	7	7	2.76	2.86	2.55	1.85	.0015	.0026	4.66	4.57	.0037
11	chocolatecroissant	21%	1	pastry	weapon	6	6	4.37	4.29	1.55	1.90	.0057	.0067	4.97	4.76	.0011
12	solderingwire	21%	2	wire	pond	4	4	4.29	4.20	1.00	1.00	.0089	.0097	4.72	4.90	.0084
13	hedgeshears	24%	2	shears	tendon	6	6	2.94	2.98	1.40	1.55	.0120	.0103	4.61	4.47	.0013

	Image ID	%Agree	Set	Word		Length		Zipf		OLD20		BG		CNC		Cosine PPMI
				C	I	C	I	C	I	C	I	C	I	C	I	
14	pouch01b	26%	1	pouch	ledge	5	5	3.36	3.38	1.05	1.10	.0069	.0051	4.50	4.72	0
15	ram	27%	2	ram	pup	3	3	3.65	3.74	1.00	1.00	.0037	.0014	4.55	4.61	.0083
16	oats	28%	1	oats	lice	4	4	3.33	3.29	1.00	1.00	.0041	.0051	4.78	4.73	.0059
17	bandage	28%	2	bandage	whisker	7	7	3.22	3.10	1.80	1.65	.0071	.0087	4.85	4.70	.0087
18	bastingbrush	28%	2	brush	stamp	5	5	4.29	4.20	1.35	1.30	.0029	.0050	4.54	4.70	.0021
19	rug01	29%	1	rug	soy	3	3	3.57	3.64	1.00	1.00	.0014	.0028	4.79	4.70	0
20	radio01	29%	1	radio	smile	5	5	4.82	4.71	1.40	1.00	.0036	.0040	4.74	4.50	.0012
21	tonfa	29%	2	baton	yeast	5	5	3.53	3.64	1.00	1.50	.0097	.0076	4.64	4.72	.0036
22	salsa	29%	1	salsa	trunk	5	5	3.82	3.93	1.20	1.15	.0038	.0025	4.70	4.71	.0010
23	smokedsalmon	29%	2	salmon	tunnel	6	6	4.34	4.23	1.25	1.65	.0058	.0039	4.81	4.82	.0007
24	handmixer01d	31%	2	mixer	wedge	5	5	3.45	3.45	1.40	1.25	.0056	.0048	4.33	4.41	.0019
25	videotape01b	31%	1	cassette	revolver	8	8	2.94	2.85	1.95	1.75	.0058	.0079	4.60	4.69	.0019
26	woodboard	31%	2	wood	ship	4	4	4.78	4.77	1.00	1.00	.0034	.0047	4.85	4.87	.0097
27	jar03	33%	2	jar	lip	3	3	3.96	3.91	1.00	1.00	.0055	.0035	5.00	4.96	.0039
28	cuttingpliers02	33%	2	pliers	beanie	6	6	2.73	2.80	1.65	1.35	.0070	.0082	4.93	4.74	.0019
29	kalashnikov	33%	2	rifle	altar	5	5	3.62	3.58	1.65	1.00	.0042	.0063	4.85	4.85	.0057
30	overalls	33%	1	overalls	mongoose	8	8	3.01	2.94	2.00	2.70	.0079	.0070	4.74	4.89	.0025
31	towel01	34%	1	towel	spine	5	5	3.87	3.91	1.30	1.00	.0077	.0090	4.86	4.88	.0090
32	branch02	36%	1	branch	powder	6	6	4.10	4.17	1.15	1.55	.0064	.0065	4.90	4.76	0
33	ribbon03a	36%	2	lace	beak	4	4	3.73	3.83	1.00	1.00	.0041	.0059	4.85	4.96	.0023

	Image ID	%Agree	Set	Word		Length		Zipf		OLD20		BG		CNC		Cosine PPMI
				C	I	C	I	C	I	C	I	C	I	C	I	
34	yarn	36%	1	yarn	twig	4	4	3.14	3.22	1.00	1.20	.0041	.0028	4.93	4.75	0
35	napkin	36%	1	napkin	weasel	6	6	3.31	3.33	1.90	1.60	.0062	.0076	4.93	4.74	.0028
36	bag	36%	1	bag	oil	3	3	4.89	4.98	1.00	1.00	.0021	.0033	4.90	4.93	.0026
37	mussel	36%	2	clam	sash	4	4	3.35	3.37	1.00	1.00	.0029	.0050	4.89	4.67	.0033
38	tray	37%	1	tray	sail	4	4	4.15	4.17	1.00	1.00	.0038	.0035	4.74	4.59	.0032
39	brainmodel	38%	1	brain	river	5	5	4.84	4.93	1.00	1.00	.0086	.0094	4.69	4.89	.0014
40	megaphone	38%	1	megaphone	billiards	9	9	2.89	2.89	2.85	2.30	.0050	.0046	4.76	4.61	.0018
41	foodprocessor	38%	2	blender	javelin	7	7	3.32	3.35	1.45	1.85	.0098	.0087	5.00	4.90	.0022
42	slide02	38%	2	slide	trail	5	5	4.17	4.27	1.15	1.10	.0036	.0038	4.48	4.46	.0078
43	turnip	38%	2	turnip	nickel	6	6	3.36	3.27	1.70	1.35	.0024	.0040	4.79	4.79	.0013
44	oyster02	38%	1	oyster	canvas	6	6	3.82	3.95	1.55	1.80	.0080	.0068	4.85	4.78	.0017
45	giftbow02b	39%	1	bow	jam	3	3	4.22	4.34	1.00	1.00	.0040	.0018	4.61	4.71	.0072
46	mask02a	39%	1	mask	pony	4	4	4.04	3.96	1.00	1.00	.0046	.0059	4.96	4.90	.0045
47	bulldozer	40%	1	bulldozer	pepperoni	9	9	2.91	2.95	2.50	2.70	.0055	.0067	4.90	5.00	0
48	iceberglettuce	41%	1	lettuce	pyramid	7	7	3.81	3.71	2.40	2.50	.0038	.0022	4.97	4.96	.0031
49	leek	42%	1	leek	moat	4	4	3.56	3.69	1.00	1.00	.0047	.0045	4.92	4.69	.0013
50	scalpel	43%	1	scalpel	tequila	7	7	3.10	3.19	1.85	2.60	.0043	.0034	4.86	4.77	0
51	pipe	43%	2	pipe	taxi	4	4	4.26	4.30	1.00	1.00	.0019	.0016	4.88	4.93	.0011
52	glassescase	44%	1	wallet	brandy	6	6	3.81	3.83	1.20	1.25	.0074	.0077	4.81	4.81	.0038
53	coaster	44%	2	tile	mast	4	4	3.56	3.51	1.00	1.00	.0068	.0080	4.68	4.92	.0057

	Image ID	%Agree	Set	Word		Length		Zipf		OLD20		BG		CNC		Cosine PPMI
				C	I	C	I	C	I	C	I	C	I	C	I	
54	lectern01	45%	2	podium	liquid	6	6	4.17	4.25	1.85	1.75	.0018	.0022	4.89	4.72	.0032
55	doorlock	46%	2	lock	rail	4	4	4.42	4.40	1.00	1.00	.0028	.0040	4.65	4.90	.0045
56	puzzle	48%	1	puzzle	sketch	6	6	3.93	3.85	1.65	1.70	.0018	.0031	4.75	4.56	.0069
57	rhinoceros02	48%	2	rhinoceros	aftershave	10	10	3.07	3.14	3.55	3.35	.0084	.0075	4.75	4.56	.0012
58	box01a	49%	1	box	sun	3	3	5.12	5.01	1.00	1.00	.0015	.0028	4.90	4.83	.0033
59	star	50%	2	star	wall	4	4	5.04	5.05	1.00	1.00	.0086	.0079	4.69	4.86	.0015
60	scanner	50%	1	scanner	bedding	7	7	3.46	3.55	1.75	1.35	.0089	.0093	4.79	4.61	0
61	mug05	50%	2	mug	wax	3	3	3.93	3.87	1.00	1.00	.0014	.0027	4.80	4.97	.0069
62	ladle02a	51%	1	ladle	tiara	5	5	3.21	3.16	1.50	1.45	.0041	.0059	4.90	4.89	.0050
63	humanskeleton	52%	1	skeleton	tortoise	8	8	3.78	3.82	2.05	2.60	.0073	.0089	4.97	4.87	.0025
64	gecko	52%	1	lizard	barley	6	6	3.73	3.61	1.60	1.00	.0038	.0051	4.68	4.59	.0009
65	boxtrailer	52%	2	trailer	receipt	7	7	3.68	3.68	1.70	2.20	.0071	.0049	4.79	4.86	.0058
66	mechanicalpencil02	53%	1	pencil	kidney	6	6	3.98	3.94	1.90	1.70	.0047	.0030	4.88	4.96	.0086
67	spatula03	54%	2	spatula	airship	7	7	2.95	2.83	2.05	2.35	.0039	.0040	4.96	4.92	.0002
68	fusilli03a	54%	1	pasta	motor	5	5	4.19	4.25	1.00	1.60	.0066	.0082	4.86	4.84	.0022
69	bracelet01	54%	2	bracelet	postcard	8	8	3.79	3.72	2.60	2.60	.0047	.0048	4.96	4.93	.0047
70	riverotter	55%	2	otter	wrist	5	5	3.80	3.84	1.00	1.15	.0090	.0073	4.86	4.93	.0042
71	grandpiano	55%	2	piano	salad	5	5	4.36	4.38	1.10	1.00	.0072	.0049	4.90	4.97	0
72	canoepaddle02	55%	2	paddle	buzzer	6	6	3.73	3.73	1.30	1.70	.0029	.0046	4.80	4.66	.0039
73	suitcase	56%	2	suitcase	pavement	8	8	3.78	3.68	2.85	2.10	.0057	.0070	4.97	4.72	.0014

	Image ID	%Agree	Set	Word		Length		Zipf		OLD20		BG		CNC		Cosine PPMI
				C	I	C	I	C	I	C	I	C	I	C	I	
74	aquarium	57%	1	aquarium	textbook	8	8	3.33	3.38	2.45	2.75	.0028	.0028	4.77	4.86	.0026
75	trombone	57%	2	trombone	mosquito	8	8	3.27	3.29	2.50	2.55	.0058	.0051	4.90	4.88	0
76	spaghetti01	57%	1	spaghetti	underwear	9	9	3.79	3.72	3.25	2.60	.0071	.0084	5.00	4.96	.0061
77	thimble	58%	2	thimble	oregano	7	7	2.98	3.00	1.80	2.15	.0106	.0096	5.00	4.81	0
78	syringe01	58%	2	syringe	mascara	7	7	3.12	3.05	1.85	1.80	.0080	.0058	4.81	4.93	0
79	antenna	59%	2	antenna	sirloin	7	7	3.01	2.95	1.95	2.70	.0087	.0067	4.75	4.66	0
80	notebook03a	59%	1	notebook	pendulum	8	8	3.32	3.30	2.75	2.70	.0044	.0049	4.92	4.69	.0001
81	cleaver01	59%	2	knife	album	5	5	4.49	4.55	1.75	1.75	.0021	.0033	4.90	4.69	.0037
82	honeydewmelon	59%	2	melon	timer	5	5	3.49	3.40	1.00	1.00	.0084	.0097	4.78	4.69	.0003
83	platypus	60%	2	platypus	campfire	8	8	2.82	2.94	2.75	2.55	.0035	.0050	4.83	4.79	.0014
84	shelf	60%	1	shelf	trout	5	5	4.02	3.96	1.50	1.00	.0108	.0087	4.96	4.72	<.0001
85	macaroni01	60%	2	macaroni	bookcase	8	8	3.08	3.02	1.95	2.70	.0068	.0044	4.97	4.93	.0014
86	apricot	61%	1	peach	valve	5	5	3.62	3.53	1.00	1.55	.0054	.0050	4.90	4.83	0
87	seaturtle	62%	1	turtle	pelvis	6	6	3.64	3.53	1.65	1.75	.0039	.0048	5.00	4.93	.0012
88	triangle	62%	1	triangle	lighting	8	8	3.93	4.00	1.85	1.35	.0072	.0086	4.52	4.38	.0037
89	vulture	62%	1	vulture	measles	7	7	3.20	3.12	1.80	1.90	.0052	.0074	4.73	4.69	.0024
90	balcony02	64%	1	balcony	seaweed	7	7	3.83	3.83	1.90	1.85	.0056	.0063	4.68	4.89	.0033
91	adjustablewrench01b	64%	2	wrench	blouse	6	6	3.15	3.08	1.60	1.75	.0076	.0078	4.93	4.96	.0048
92	cane	64%	2	cane	reef	4	4	3.75	3.79	1.00	1.00	.0107	.0087	4.87	4.70	.0053
93	shield02	64%	2	shield	packet	6	6	3.80	3.90	1.70	1.45	.0051	.0036	4.66	4.46	0

	Image ID	%Agree	Set	Word		Length		Zipf		OLD20		BG		CNC		Cosine PPMI
				C	I	C	I	C	I	C	I	C	I	C	I	
94	tank	64%	1	tank	seed	4	4	4.34	4.24	1.00	1.00	.0086	.0071	4.80	4.71	.0066
95	straw	66%	2	straw	badge	5	5	4.17	4.06	1.00	1.00	.0047	.0025	4.77	4.93	.0020
96	pickle01a	66%	2	pickle	magnet	6	6	3.66	3.70	1.10	1.70	.0034	.0039	4.64	4.70	.0081
97	axe01	67%	1	axe	rum	3	3	3.85	3.91	1.00	1.00	.0001	.0010	5.00	4.93	.0023
98	boat	67%	1	boat	card	4	4	4.89	4.89	1.00	1.00	.0044	.0059	4.93	4.90	.0093
99	bowl01	67%	1	bowl	neck	4	4	4.69	4.65	1.00	1.00	.0027	.0044	4.87	5.00	.0063
100	plunger02	67%	2	plunger	caribou	7	7	3.03	2.93	1.65	1.95	.0072	.0073	4.96	4.92	.0069
101	panda	67%	1	panda	lever	5	5	3.73	3.66	1.00	1.00	.0091	.0100	4.75	4.77	.0008
102	toothpick02	67%	2	toothpick	periscope	9	9	2.79	2.81	3.35	2.65	.0090	.0069	4.93	4.78	.0011
103	kettle01	67%	2	kettle	picnic	6	6	4.02	4.04	1.45	1.90	.0041	.0027	4.75	4.83	0
104	lime	67%	2	lime	swan	4	4	4.09	3.98	1.00	1.00	.0061	.0083	4.96	4.96	.0031
105	razor01	68%	1	razor	strap	5	5	3.69	3.62	1.75	1.00	.0038	.0049	4.90	4.79	.0021
106	sailboat	69%	1	sailboat	knapsack	8	8	2.08	2.04	2.50	3.00	.0034	.0020	4.89	4.90	.0089
107	ribbon04	69%	1	ribbon	bunker	6	6	3.58	3.63	1.85	1.30	.0047	.0065	4.89	4.79	.0015
108	barn	69%	2	barn	menu	4	4	4.32	4.36	1.00	1.00	.0048	.0070	4.79	4.67	.0009
109	moon	69%	2	moon	seat	4	4	4.74	4.78	1.00	1.00	.0072	.0088	4.90	4.78	.0001
110	parrot01	69%	2	parrot	sleeve	6	6	3.84	3.88	1.65	1.70	.0053	.0054	5.00	4.84	.0016
111	bacon	71%	1	bacon	photo	5	5	4.34	4.42	1.00	1.55	.0067	.0063	4.90	4.93	0
112	americangoldfinch	71%	2	bird	cake	4	4	4.85	4.81	1.00	1.00	.0021	.0039	5.00	4.81	.0035
113	cheetah	71%	1	cheetah	stopper	7	7	3.45	3.39	2.20	1.55	.0090	.0083	4.70	4.83	0

	Image ID	%Agree	Set	Word		Length		Zipf		OLD20		BG		CNC		Cosine PPMI
				C	I	C	I	C	I	C	I	C	I	C	I	
114	seagull	71%	2	seagull	apricot	7	7	3.30	3.29	2.50	2.40	.0053	.0045	5.00	4.97	.0008
115	nail	72%	2	nail	sofa	4	4	4.18	4.22	1.00	1.00	.0034	.0047	4.93	4.90	.0079
116	starfish01	72%	2	starfish	armchair	8	8	3.27	3.31	2.15	2.80	.0065	.0056	4.90	5.00	.0054
117	pill	72%	1	pill	knot	4	4	3.81	3.74	1.00	1.05	.0050	.0045	4.72	4.87	.0006
118	acorn	73%	1	acorn	bugle	5	5	3.13	3.01	1.65	1.25	.0056	.0033	4.96	4.84	.0065
119	shorts01	74%	1	shorts	needle	6	6	3.82	3.93	1.35	1.55	.0052	.0058	4.82	4.93	.0018
120	tripod01	74%	1	tripod	seesaw	6	6	3.04	2.97	1.85	1.95	.0029	.0053	4.72	4.92	0
121	cabbage	74%	2	cabbage	uniform	7	7	4.07	4.16	1.65	2.00	.0027	.0043	4.75	4.67	.0053
122	raccoon	74%	2	raccoon	notepad	7	7	2.57	2.55	2.45	2.80	.0055	.0046	4.67	4.70	.0004
123	dormer	76%	1	window	letter	6	6	4.84	4.85	1.40	1.00	.0106	.0087	4.86	4.70	.0094
124	volleyball	76%	1	volleyball	chimpanzee	10	10	3.31	3.19	3.80	3.70	.0050	.0052	4.93	4.96	0
125	cocktailshrimp02	76%	2	shrimp	tablet	6	6	3.63	3.54	1.80	1.65	.0030	.0045	4.80	4.82	.0042
126	bowrake	76%	1	rake	yolk	4	4	3.40	3.52	1.00	1.20	.0035	.0040	4.84	4.78	.0043
127	tulip02	76%	1	tulip	llama	5	5	3.21	3.12	1.70	1.60	.0031	.0054	5.00	4.78	.0022
128	tie02	79%	1	tie	map	3	3	4.58	4.52	1.00	1.00	.0051	.0031	4.81	4.93	.0008
129	popcorn	79%	1	popcorn	luggage	7	7	3.68	3.61	2.60	2.55	.0038	.0017	5.00	4.83	0
130	pigeon	79%	1	pigeon	muscle	6	6	4.03	4.11	1.70	1.80	.0048	.0034	4.71	4.50	.0027
131	honeybee	79%	1	bee	lid	3	3	4.19	4.16	1.00	1.00	.0063	.0044	4.88	4.96	0
132	callbell	79%	2	bell	oven	4	4	4.54	4.54	1.00	1.00	.0073	.0079	4.96	4.97	.0066
133	teapot	79%	1	teapot	mousse	6	6	3.78	3.76	1.90	1.35	.0054	.0076	4.96	4.83	.0039

	Image ID	%Agree	Set	Word		Length		Zipf		OLD20		BG		CNC		Cosine PPMI
				C	I	C	I	C	I	C	I	C	I	C	I	
134	rope03	79%	1	rope	text	4	4	4.30	4.40	1.00	1.10	.0040	.0035	4.93	4.93	0
135	marble	80%	2	marble	puppet	6	6	3.86	3.77	1.50	1.70	.0051	.0027	4.85	4.64	.0094
136	boot02b	82%	2	boot	page	4	4	4.43	4.52	1.00	1.00	.0043	.0028	4.96	4.90	.0009
137	plum01	82%	1	plum	ramp	4	4	3.79	3.67	1.00	1.00	.0016	.0030	4.85	4.69	.0047
138	tampon	82%	1	tampon	poncho	6	6	2.30	2.41	1.80	1.65	.0051	.0059	4.86	4.97	.0076
139	slipper01b	82%	2	slipper	warship	7	7	3.19	3.09	1.40	1.85	.0054	.0056	4.86	4.86	0
140	chalk	82%	2	chalk	organ	5	5	3.87	3.99	1.30	1.00	.0077	.0080	4.90	4.77	.0018
141	banjo	83%	2	banjo	scalp	5	5	3.31	3.28	1.45	1.35	.0057	.0040	4.90	4.82	.0050
142	peanut01	83%	2	peanut	bumper	6	6	3.65	3.53	1.95	1.40	.0078	.0058	4.89	4.96	.0091
143	pillow01a	84%	2	pillow	beetle	6	6	3.73	3.72	1.60	1.60	.0050	.0053	5.00	4.83	.0068
144	cigar	85%	2	cigar	stump	5	5	3.59	3.52	1.75	1.25	.0041	.0037	4.93	4.78	0
145	jellyfish	86%	2	jellyfish	sunflower	9	9	3.56	3.55	3.10	2.95	.0049	.0054	4.93	4.80	.0096
146	calendar	86%	2	calendar	medicine	8	8	4.33	4.31	2.30	2.10	.0085	.0084	4.62	4.79	.0017
147	bull	86%	1	bull	cave	4	4	4.28	4.19	1.00	1.00	.0052	.0064	4.85	4.96	.0058
148	daddylonglegs	86%	1	spider	tongue	6	6	4.24	4.36	1.25	1.75	.0059	.0081	4.97	4.93	.0083
149	chimney	86%	2	chimney	bicycle	7	7	3.90	3.92	1.85	2.40	.0047	.0027	5.00	4.89	.0098
150	ashtray01	87%	2	ashtray	brownie	7	7	3.20	3.29	2.30	1.75	.0043	.0033	4.97	4.82	.0055
151	binoculars01b	87%	1	binoculars	ammunition	10	10	3.59	3.61	3.45	3.00	.0065	.0057	5.00	4.88	.0099
152	baseball01a	87%	2	baseball	cinnamon	8	8	3.74	3.78	2.55	2.55	.0057	.0072	4.86	4.85	0
153	broom01	87%	1	broom	algae	5	5	3.56	3.44	1.15	1.60	.0045	.0026	4.89	4.93	.0051

	Image ID	%Agree	Set	Word		Length		Zipf		OLD20		BG		CNC		Cosine PPMI
				C	I	C	I	C	I	C	I	C	I	C	I	
154	balloon01b	87%	1	balloon	stomach	7	7	4.25	4.28	1.65	1.95	.0073	.0072	4.92	4.89	.0071
155	avocado01	87%	1	avocado	sparrow	7	7	3.25	3.38	2.55	1.80	.0031	.0046	4.89	4.85	.0025
156	sock01a	87%	2	sock	tuna	4	4	3.77	3.76	1.00	1.00	.0029	.0025	4.91	4.89	.0047
157	jeans01	88%	1	jeans	wagon	5	5	3.84	3.73	1.30	1.60	.0078	.0065	5.00	4.89	.0012
158	nose	88%	2	nose	mail	4	4	4.72	4.63	1.00	1.00	.0057	.0042	4.89	4.69	.0017
159	knee	88%	2	knee	soil	4	4	4.26	4.35	1.35	1.00	.0048	.0039	5.00	4.87	.0075
160	stool01	88%	2	stool	weeds	5	5	3.71	3.66	1.05	1.00	.0078	.0054	4.90	4.83	0
161	jeep	88%	1	jeep	wick	4	4	3.17	3.16	1.00	1.00	.0023	.0039	4.80	4.69	.0004
162	cannon	88%	2	cannon	throat	6	6	4.08	4.16	1.15	1.70	.0092	.0116	4.79	4.97	.0022
163	ostrich	88%	2	ostrich	shuttle	7	7	3.52	3.58	2.10	1.80	.0053	.0036	4.71	4.63	.0077
164	porcupine	88%	1	porcupine	lawnmower	9	9	3.06	3.11	3.25	3.45	.0064	.0052	5.00	4.97	.0023
165	arrow02	90%	2	arrow	jewel	5	5	3.78	3.76	1.00	1.75	.0059	.0035	4.97	4.96	.0006
166	tricycle	90%	2	tricycle	songbird	8	8	2.73	2.75	2.60	2.80	.0033	.0053	4.68	4.59	.0062
167	sponge01	90%	2	sponge	timber	6	6	4.12	4.05	1.45	1.40	.0068	.0075	5.00	4.90	.0002
168	celery	92%	1	celery	tattoo	6	6	3.66	3.78	1.90	1.85	.0082	.0067	4.80	4.71	.0039
169	violin	92%	1	violin	burger	6	6	3.82	3.90	1.75	1.15	.0081	.0065	4.96	4.93	.0014
170	iron01b	92%	1	iron	soup	4	4	4.52	4.41	1.00	1.00	.0078	.0086	4.59	4.72	.0060
171	lamp04a	92%	1	lamp	wool	4	4	4.09	4.11	1.00	1.00	.0030	.0036	4.97	4.86	.0093
172	scarf	92%	2	scarf	patio	5	5	3.76	3.73	1.05	1.35	.0043	.0058	4.97	4.89	.0026
173	microscope	92%	2	microscope	spacecraft	10	10	3.56	3.46	2.50	3.25	.0035	.0025	5.00	4.80	.0083

	Image ID	%Agree	Set	Word		Length		Zipf		OLD20		BG		CNC		Cosine PPMI
				C	I	C	I	C	I	C	I	C	I	C	I	
174	rice	92%	1	rice	bomb	4	4	4.42	4.49	1.00	1.00	.0050	.0031	4.86	4.84	.0075
175	rooster	93%	1	rooster	serpent	7	7	3.13	3.16	1.50	1.70	.0087	.0088	4.75	4.97	.0008
176	beaver	93%	1	beaver	shrine	6	6	3.50	3.53	1.00	1.55	.0098	.0088	4.68	4.47	.0097
177	trophy01	93%	2	trophy	jacket	6	6	4.37	4.29	1.90	1.40	.0025	.0032	4.89	4.86	.0032
178	cactus	93%	2	cactus	poodle	6	6	3.35	3.27	1.70	1.45	.0037	.0035	5.00	4.89	0
179	snowboard	95%	2	snowboard	amplifier	9	9	2.84	2.73	2.65	2.65	.0035	.0051	4.86	4.79	.0076
180	potato02b	95%	1	potato	ticket	6	6	4.44	4.51	1.60	1.35	.0071	.0048	4.85	4.70	.0086
181	apple07	95%	1	apple	penny	5	5	4.58	4.49	1.40	1.00	.0034	.0044	5.00	4.83	.0080
182	apron	95%	2	apron	lager	5	5	3.48	3.56	1.05	1.00	.0062	.0075	4.87	4.64	.0001
183	cigarette	95%	2	cigarette	porcelain	9	9	4.11	4.10	2.80	2.90	.0065	.0071	4.88	4.63	.0091
184	skunk	95%	1	skunk	quail	5	5	3.36	3.48	1.55	1.45	.0016	.0024	4.88	4.65	.0054
185	barnowl	95%	2	owl	jug	3	3	4.07	4.06	1.00	1.00	.0026	.0016	4.93	4.96	.0095
186	lipstick02a	95%	1	lipstick	cardigan	8	8	3.62	3.50	2.30	1.90	.0047	.0064	4.90	4.96	.0096
187	brick	95%	1	brick	robot	5	5	4.18	4.09	1.00	1.60	.0036	.0040	4.83	4.65	.0017
188	leaf02a	97%	2	leaf	pork	4	4	4.29	4.39	1.00	1.00	.0059	.0048	5.00	4.79	0
189	carrot01	97%	2	carrot	tissue	6	6	4.08	3.97	1.40	1.75	.0059	.0051	5.00	4.93	.0053
190	kite	98%	2	kite	cart	4	4	3.89	3.77	1.00	1.00	.0084	.0064	5.00	4.89	.0004
191	locker	98%	1	locker	manual	6	6	3.60	3.70	1.00	1.75	.0064	.0069	4.67	4.45	.0048
192	pumpkin	98%	2	pumpkin	trolley	7	7	3.79	3.82	1.70	1.70	.0052	.0055	4.90	4.73	.0025
193	zebra	98%	2	zebra	snail	5	5	3.69	3.69	1.80	1.45	.0016	.0026	4.86	4.93	.0062

	Image ID	%Agree	Set	Word		Length		Zipf		OLD20		BG		CNC		Cosine PPMI
				C	I	C	I	C	I	C	I	C	I	C	I	
194	kangaroo	98%	1	kangaroo	lemonade	8	8	3.62	3.57	2.75	2.70	.0077	.0055	4.86	4.83	.0058
195	squirrel	100%	1	squirrel	passport	8	8	3.94	4.01	2.10	2.25	.0045	.0045	4.89	5.00	.0051
196	mushroom01	100%	2	mushroom	carriage	8	8	3.87	3.98	2.60	1.90	.0040	.0044	4.83	4.86	.0003
197	pear01	100%	1	pear	lung	4	4	3.83	3.81	1.00	1.00	.0078	.0055	4.93	4.82	.0050
198	snowman	100%	1	snowman	pancake	7	7	3.52	3.48	1.90	2.05	.0060	.0059	4.64	4.86	.0054
199	onion	100%	1	onion	torch	5	5	4.28	4.21	1.70	1.30	.0086	.0073	4.86	4.76	.0013
200	toothbrush03b	100%	2	toothbrush	cheesecake	10	10	3.48	3.54	3.80	3.45	.0084	.0085	5.00	4.97	.0040

B Behavioural Validation Results

To validate the stimulus generation method for the picture-word stimuli, we ran a behavioural experiment using a stimulus set generated from a very similar pipeline to that described in the manuscript. The only differences in the pipeline were that (a) Zipf frequency was controlled within $\pm.2$, (b) Levenshtein distance was not maximised, (c) OLD20 was not controlled for, and (d) the split into stimulus Sets 1 and 2 was optimised from only 20,000 iterations. The stimuli generated for the validation experiment varied in predictability from 12 to 100%. The procedure was also identical to that described in the Procedure section of the manuscript, except that participants could respond as soon as the word was presented, rather than 1 second after presentation, and the word did not change colour. Participants comprised 35 monolingual native English speakers (15 female, 19 male, 1 non-binary) who were not diagnosed with any reading disorder. Age varied from 18 to 26 years (M=21.4, *SD*=2.05), and all participants reported being right-handed with normal or corrected-to-normal vision. Trials were excluded if response times (RTs) were less than 250 ms or more than 2000 ms. The logic for the validation experiment was as follows: assuming the stimulus pipeline produces suitably controlled stimuli, increased predictability should facilitate task performance for congruent trials and have either no effect or a minimal effect on performance for incongruent trials.

We modelled the RT data with a shifted log-normal distribution. This allowed us to describe changes in the means (μ) and standard deviations (σ) of log-transformed RTs, while also modelling changes in shift (δ). To model the validation experiment data, we fit a Bayesian mixed-effects model estimating the same fixed and maximal random effects structure for each parameter (μ, σ, δ) of the shifted log-normal distribution. This was achieved using the *brms* package for R (Bürkner, 2017), a high-level interface for STAN (STAN Development Team, 2023). This model estimated the plausibility of population values for each parameter of the shifted log-normal distribution as a function of the maximal hierarchical structure justified by the experiment's design. The parameter of μ

was modelled with an identity link function, while σ and δ were modelled with log link functions. The same predictors and random effects structure were used for each parameter as described for the EEG experiment, though with a key difference being that predictability was normalised between 12% and 100% rather between than 7% and 100%, due to different minima in the experiments' stimuli. The full formula, in *brms* syntax, was specified as:

```
rt ~ 1 + congruency * predictability +
        (1 + congruency * predictability | subject_id) +
        (1 + congruency | image_id) +
        (1 | word_id),
sigma ~ 1 + congruency * predictability +
        (1 + congruency * predictability | subject_id) +
        (1 + congruency | image_id) +
        (1 | word_id),
ndt ~ 1 + congruency * predictability +
        (1 + congruency * predictability | subject_id) +
        (1 + congruency | image_id) +
        (1 | word_id)
```

Prior distributions were specified to be broad enough as to be uninformative but constrained to cover plausible values for response time distributions for a cognitive task (Figure 2). Fixed effects' slopes' prior distributions were drawn from $N(0, 2.5)$ and fixed effects' intercepts' prior distributions from $N(0, 7.5)$. The prior distributions for the standard deviations of random effects were specified as student's t distributions centred on zero, with 3 degrees of freedom and a scale parameter of 2. The model was fit with 5 Markov chains, each with 25,000 iterations (17,500 warm-up and 7,500 sampling). The *adapt_delta* parameter was set to .99. The densities of the posterior distributions, relative to those of the priors, are shown in Figure 2.

The results from the shifted log-normal model showed the expected effects, with predictability leading to faster responses for congruent trials, but having weak effects on incongruent trials (Figure 3). It also demonstrated that when predictability is low, response times show similar central tendency for congruent and incongruent trials though a larger spread in the distribution for congruent trials. When predictability is high, on the

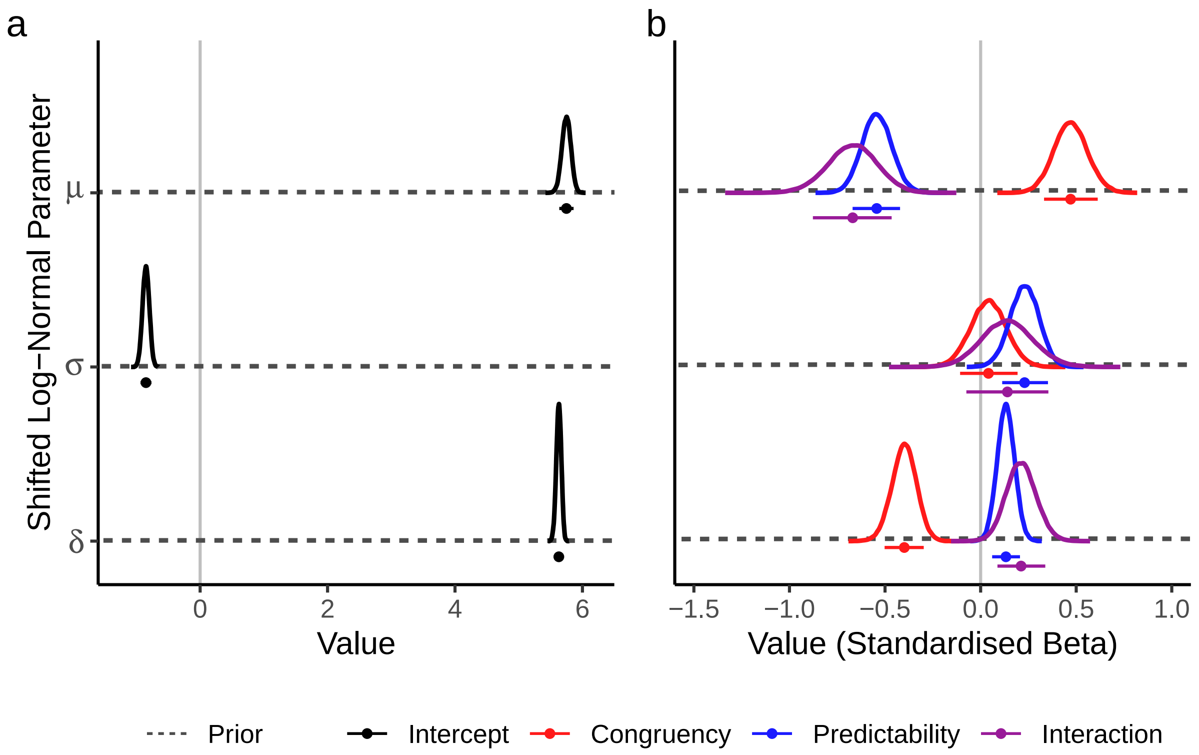

Figure 2

*Prior (dashed lines) and posterior distributions (solid lines) for all fixed effects estimated in the Bayesian shifted log-normal model, for the three distribution parameters. Panels show distributions for (**a**) intercept parameters, and (**b**) slope parameters. For both panels, points below distributions' densities depict median posterior estimates, while the whiskers show the extents of 89% highest density intervals (HDIs).*

other hand, the difference is mostly due to changes in shift, whereas other features of the distribution are very similar.

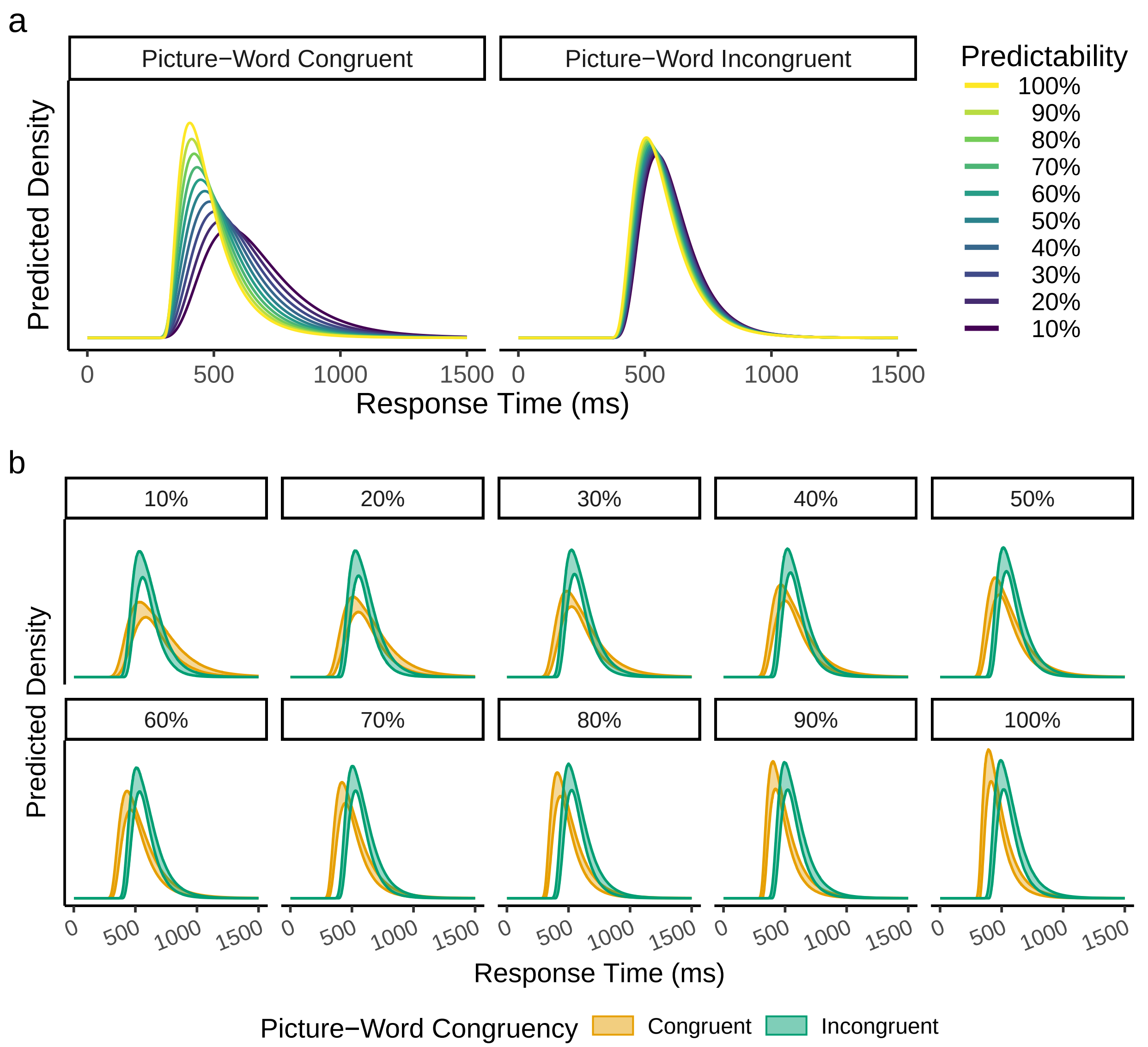

Figure 3

*Fixed effect predictions of RT distributions in the behavioural validation experiment for the picture-word stimuli. Predictions of RT distributions, derived from the shifted log-normal model, are shown for congruent and incongruent trials for values of percentage of name agreement, from 10 to 100% in steps of 10. The two panels show the same results but highlight (**a**) the effect of predictability for picture-congruent and picture-incongruent words, and (**b**) the effect of picture-word congruency at different values of predictability, showing the degree of certainty in the predictions with the 89% HDIs of the predictions from all posterior samples. Density is scaled consistently across panels.*

C Localiser Task Stimuli

To generate the localiser stimuli, a large list of suitable words (N=27,332) was identified by subsetting the word prevalence norms of Brysbaert et al. (2019) to only contain words known by at least 90% of participants and which were not selected for the main experiment. A representative sample (N=100) of this list was generated by maximising distributional overlap (Pastore & Calcagnì, 2019), between the sample and the full list of candidates, on 13 variables where observations were available: (1) word prevalence (Brysbaert et al., 2019); (2) length (number of characters); (3) word frequency in Zipf in SUBTLEX-UK (van Heuven et al., 2014); (4) part of speech according to SUBTLEX-UK; (5) character bigram probability calculated from SUBTLEX-UK; (6) OLD20 (Yarkoni et al., 2008) calculated from the LexOPS dataset (Taylor et al., 2020); (7) concreteness (Brysbaert et al., 2014); (8) age of acquisition (Kuperman et al., 2012); (9, 10) average lexical decision response time (RT) and accuracy according to the British Lexicon Project (Keuleers et al., 2012); and (11, 12, 13) the emotion ratings of valence, arousal, and dominance (Warriner et al., 2013). Similarity in the categorical variable of part of speech was maximised with dummy-coded variables (0 or 1 for absence or presence of a category, respectively). Distributional similarity across all variables was maximised by selecting from 500,000 random samples the sample with the highest total distributional overlap with the full list of possible words. Distributions of the selected sample of words are summarised in **Figure 4**. The full list of stimuli for the localiser task is presented in **Table 2**.

The false-font strings consisted of characters from the Brussels Artificial Character Set (BACS; Vidal et al., 2017) in BACS2serif font. In this way, we had an item-wise false-font match to each word, where every Courier New character in the word stimuli is replaced with a BACS character matched in the number of strokes, junctions, terminations, and serifs. The phase-shuffled stimuli were generated by using a Fourier transformation to extract the phase and amplitude from the word images. Phase values were randomly shuffled (i.e., permuted), such that the overall distribution of phase could be preserved,

Figure 4

Distributions of key variables illustrate the similarity between the selected localiser stimuli words (sample) and the list of words from which they were drawn (population).

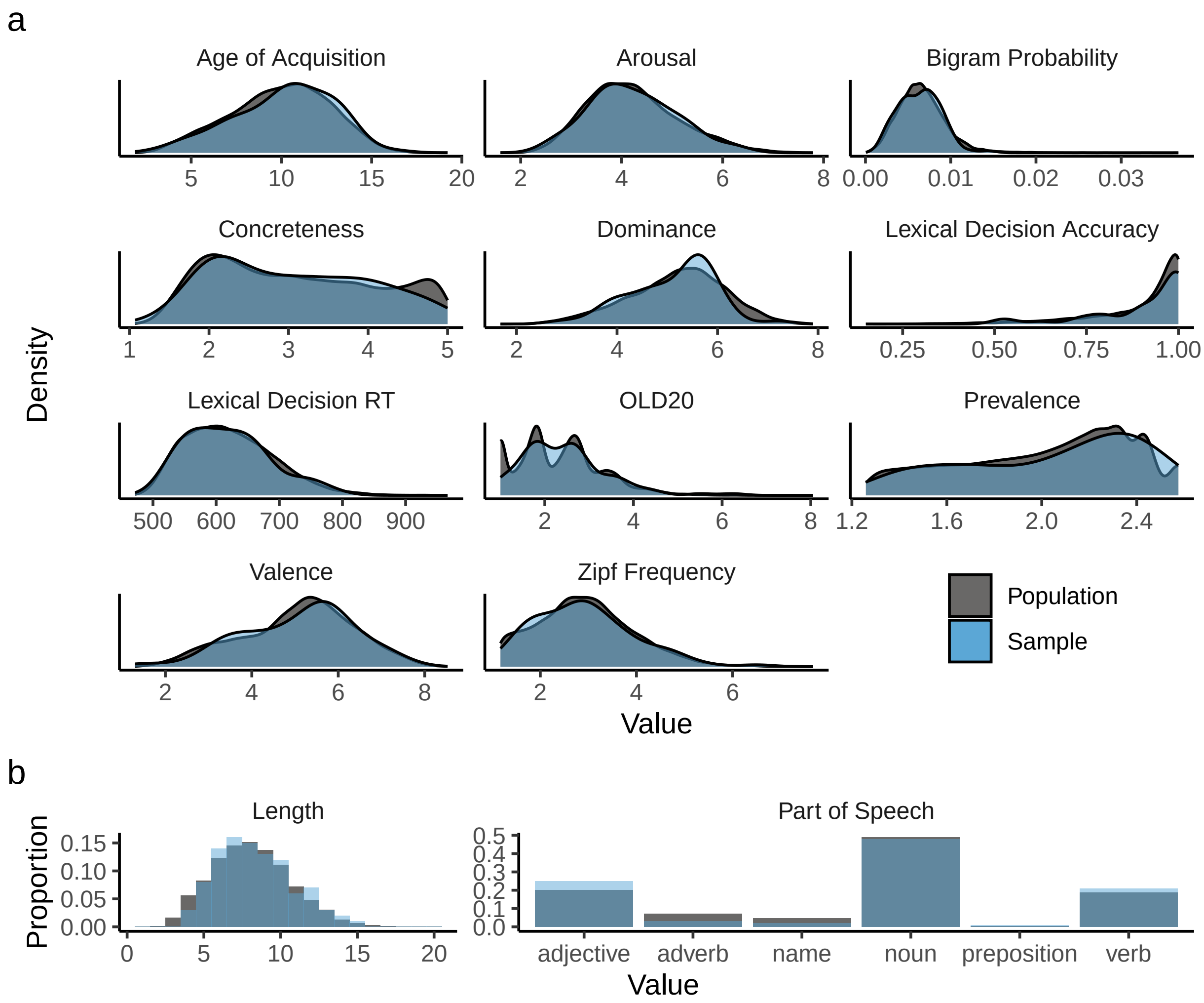

Panel **a** shows distributional similarity of continuous variables. Panel **b** shows similarity in length (all integer values) as a histogram showing proportions, and the similarity in the counts of each part of speech category as a bar plot of proportions. Only the part of speech categories which were present in the sample are shown. No members of less common part of speech categories, such as determiner or number, were selected in the sample.

while amplitude values were unchanged. An inverse Fourier transformation was then used to generate a new image with the original amplitude values, but with phase randomly shuffled. To prevent phase shuffling from producing noticeably large changes in contrast, the phase shuffling was done on a version of the word image with 50% of the original

contrast. After the inverse Fourier transformation, the contrast of the generated phase-shuffled image was readjusted to equal that of the original word image. To avoid repeating the same stimuli across participants more than necessary, unique phase-shuffled images were generated for each trial, for each participant.

Versions of the localiser task's stimuli were also created in green, to signal the participant to respond. For words and nonwords, this was done by simply changing the font colour to green. To preserve image intensity, the colour of phase-shuffled images was changed by altering pixels in the following way. For pixels in which the value in the green channel was less than 50% of the maximum intensity (i.e., the intensity of all channels in the grey background), values in red and blue channels were altered to equal the value in the green channel for that pixel. For all other pixels, the values in red and blue channels were set to 50% of the maximum intensity.

Table 2

All word stimuli for the localiser task, and associated values on variables that were matched distribution-wise. False-font strings and phase-shuffled images are not presented here; false-font strings were just the words in BACS2serif font, while a unique phase-shuffled image was generated for each trial. The columns are as follows: Word *= words presented in the task;* Length *= number of characters;* Zipf *= Zipf frequency in SUBTLEX-UK;* PREV *= word prevalence values in Brysbaert et al. (2019);* OLD20 *= OLD20 values in the LexOPS dataset;* BG *= mean character bigram probabilities in SUBTLEX-UK;* PoS *= dominant part of speech in SUBTLEX-UK;* CNC *= mean concreteness ratings in Brysbaert et al. (2014);* AoA *= mean age of acquisition ratings in Kuperman et al. (2012);* VAL, AROU, *and* DOM *= mean valence, arousal, and dominance ratings, respectively, from Warriner et al. (2013);* LDT RT *and* LDT Acc *= average response times (in ms) and accuracies in lexical decision, from the BLP. Rows are numbered for ease of reference.*

	Word	Length	Zipf	PREV	OLD20	BG	PoS	CNC	AoA	VAL	AROU	DOM	LDT RT	LDT Acc
1	tracker	7	3.12	2.58	1.45	.0062	noun	3.89	9.61	4.87	4.59	5.00	583.21	.98
2	tablespoonful	13	1.97	1.40	5.30	.0045	adjective	4.24	7.58	-	-	-	-	-
3	curricular	10	2.50	1.61	2.85	.0042	adjective	2.77	10.10	-	-	-	-	-
4	sheathed	8	1.74	1.45	1.95	.0195	verb	3.04	-	-	-	-	699.78	.68
5	wasabi	6	2.74	1.74	2.00	.0041	noun	4.67	13.95	-	-	-	-	-
6	persecute	9	2.43	1.80	2.65	.0068	verb	2.53	10.06	3.11	5.11	4.09	-	-
7	enlarge	7	2.70	2.16	2.35	.0053	verb	3.17	8.26	5.33	3.87	5.89	568.70	.95
8	harvester	9	3.15	2.12	2.60	.0107	noun	4.21	9.53	-	-	-	-	-
9	campaign	8	4.90	2.44	2.20	.0027	noun	3.00	12.55	4.55	3.50	5.14	561.37	.98
10	menacingly	10	2.20	1.79	3.25	.0078	adverb	1.93	-	-	-	-	-	-
11	footwork	8	3.40	2.13	2.15	.0044	noun	3.32	10.63	5.74	3.96	5.58	680.59	.88
12	respective	10	3.20	2.10	2.65	.0067	adjective	1.79	10.78	5.90	3.76	6.42	-	-
13	layperson	9	1.65	1.35	2.85	.0068	noun	3.44	13.74	-	-	-	-	-

	Word	Length	Zipf	PREV	OLD20	BG	PoS	CNC	AoA	VAL	AROU	DOM	LDT RT	LDT Acc
14	microcomputer	13	1.30	1.82	4.45	.0055	noun	4.55	13.89	-	-	-	-	-
15	flatterer	9	2.32	1.32	1.85	.0104	noun	2.89	12.44	-	-	-	-	-
16	chilled	7	3.63	2.35	1.75	.0074	verb	3.22	-	-	-	-	566.50	1.00
17	blackheads	10	1.93	2.07	2.35	.0065	noun	4.79	-	-	-	-	742.83	.97
18	fortunate	9	4.06	2.24	2.50	.0056	adjective	2.04	10.17	7.33	3.81	5.83	635.46	.95
19	screeching	10	2.81	2.24	2.55	.0092	verb	3.71	-	-	-	-	621.72	.93
20	chimp	5	3.42	2.23	1.35	.0048	noun	4.96	7.17	6.00	3.80	4.95	605.63	.88
21	payroll	7	3.10	2.43	2.40	.0042	noun	3.70	12.79	6.19	3.82	5.11	632.25	.97
22	seer	4	2.48	1.26	1.00	.0110	noun	-	10.56	5.35	3.77	5.41	752.86	.53
23	coexist	7	2.00	1.99	2.45	.0053	verb	2.25	11.56	5.95	3.48	5.92	-	-
24	smelly	6	3.87	2.43	1.45	.0057	adjective	3.07	4.32	2.68	5.43	4.00	533.24	1.00
25	discouraging	12	2.69	2.33	3.25	.0084	verb	1.83	9.11	2.89	4.17	4.22	-	-
26	exotic	6	4.10	2.43	1.85	.0039	adjective	2.11	10.42	7.55	6.90	5.65	-	-
27	snow	4	4.79	2.33	1.00	.0040	noun	4.85	4.11	6.78	4.57	5.62	506.10	1.00
28	takeoff	7	2.84	1.92	2.45	.0035	noun	3.41	7.35	5.50	3.77	5.11	-	-
29	milkman	7	3.08	1.98	1.90	.0054	noun	4.61	6.37	5.75	2.73	5.54	626.19	1.00
30	intelligent	11	4.09	2.58	3.15	.0094	adjective	2.46	8.28	7.60	5.67	6.77	-	-
31	creak	5	2.69	1.40	1.30	.0078	verb	3.61	8.10	4.68	4.40	4.61	599.59	.85
32	punchy	6	3.03	1.51	1.55	.0024	adjective	2.21	13.18	4.78	4.32	3.96	657.00	.76
33	glutinous	9	2.09	1.53	2.70	.0089	adjective	2.62	14.32	-	-	-	-	-
34	monsieur	8	3.70	1.35	2.75	.0046	noun	3.54	10.12	5.50	3.30	5.89	-	-
35	sympathetic	11	3.70	2.58	3.50	.0105	adjective	1.77	9.39	6.67	3.29	6.30	-	-

	Word	Length	Zipf	PREV	OLD20	BG	PoS	CNC	AoA	VAL	AROU	DOM	LDT RT	LDT Acc
36	neurotoxin	10	1.95	1.72	3.10	.0071	noun	3.12	13.58	-	-	-	-	-
37	singular	8	3.00	2.27	2.45	.0086	adjective	2.21	9.80	4.89	3.12	5.24	-	-
38	snip	4	3.64	2.00	1.00	.0012	noun	3.68	7.24	4.32	4.74	4.95	569.42	.95
39	bewildered	10	3.14	2.43	3.30	.0080	verb	1.80	11.63	4.32	4.57	4.42	-	-
40	devote	6	3.16	2.03	1.55	.0045	verb	2.00	9.58	5.53	4.05	7.05	600.51	.97
41	handily	7	2.30	1.62	1.90	.0101	adverb	2.08	-	-	-	-	-	-
42	orally	6	2.41	2.23	1.90	.0076	adverb	3.00	-	-	-	-	-	-
43	prerecorded	11	1.60	2.10	3.45	.0096	verb	2.58	10.22	-	-	-	-	-
44	yodel	5	3.11	1.49	1.55	.0054	name	4.20	8.16	6.10	3.33	5.90	703.75	.50
45	impertinently	13	1.17	1.38	3.90	.0082	adverb	-	-	-	-	-	-	-
46	vacation	8	3.36	2.58	1.85	.0063	noun	3.14	5.22	8.53	5.22	7.11	-	-
47	extravagance	12	2.86	2.20	3.85	.0038	noun	1.73	10.74	5.74	5.40	5.79	-	-
48	thud	4	3.01	2.26	1.00	.0139	noun	3.20	8.06	4.24	5.05	4.52	582.36	.83
49	forewarn	8	1.74	1.90	2.10	.0076	verb	2.20	11.16	-	-	-	703.91	.66
50	fatherhood	10	2.73	2.44	3.20	.0130	noun	2.76	8.50	6.77	4.57	5.61	-	-
51	correlate	9	2.20	2.04	2.60	.0083	verb	1.63	13.35	-	-	-	-	-
52	watercraft	10	1.54	1.61	2.90	.0056	noun	-	-	-	-	-	-	-
53	sunk	4	3.73	2.43	1.00	.0026	verb	3.46	-	-	-	-	611.78	.93
54	flawlessness	12	1.39	1.58	3.30	.0042	noun	2.16	-	-	-	-	-	-
55	tranquilizer	12	1.47	2.02	2.30	.0054	noun	4.55	11.58	4.86	3.12	4.85	-	-
56	pituitary	9	2.47	1.52	3.70	.0065	adjective	3.33	13.06	4.79	4.40	4.91	-	-
57	courtside	9	1.81	2.00	2.85	.0059	noun	3.65	12.32	6.00	4.24	6.00	-	-

	Word	Length	Zipf	PREV	OLD20	BG	PoS	CNC	AoA	VAL	AROU	DOM	LDT RT	LDT Acc
58	wicked	6	4.16	2.33	1.15	.0047	adjective	2.11	8.33	2.63	5.86	3.61	579.31	.93
59	regard	6	4.19	2.24	1.55	.0067	noun	1.79	10.20	5.70	3.39	6.38	545.31	.98
60	infidelity	10	2.71	2.33	3.55	.0072	noun	2.07	13.89	2.10	5.70	3.86	-	-
61	bumping	7	3.29	2.34	1.55	.0074	verb	4.00	-	-	-	-	660.94	.97
62	cannibal	8	2.60	2.31	2.45	.0058	adjective	3.82	9.11	2.90	6.10	3.20	-	-
63	texting	7	3.51	2.58	1.80	.0093	verb	4.23	-	-	-	-	-	-
64	apache	6	3.15	1.75	1.75	.0091	name	3.88	10.50	5.20	3.70	4.95	747.23	.68
65	generational	12	2.98	1.88	2.90	.0084	adjective	1.96	12.68	-	-	-	-	-
66	squint	6	2.79	2.33	1.75	.0075	noun	4.30	8.05	4.40	3.71	4.62	586.76	1.00
67	torture	7	4.00	2.43	1.80	.0089	verb	3.59	10.70	1.40	5.09	2.76	530.51	1.00
68	shattering	10	3.10	2.32	1.75	.0115	verb	3.43	8.00	3.67	5.00	4.63	-	-
69	freckled	8	1.30	2.43	1.90	.0061	adjective	3.86	6.58	-	-	-	645.19	.98
70	perversion	10	2.35	2.07	2.70	.0087	noun	2.04	13.11	3.55	5.48	3.85	-	-
71	shag	4	3.37	2.00	1.00	.0073	noun	3.15	10.53	5.38	4.95	4.86	546.18	.98
72	stifle	6	2.68	1.97	1.70	.0058	verb	2.59	10.26	-	-	-	659.20	.82
73	syllable	8	2.89	2.25	2.00	.0038	adjective	3.26	8.10	4.95	2.50	5.70	-	-
74	ionic	5	2.50	1.79	1.40	.0063	adjective	2.14	14.19	-	-	-	-	-
75	explicable	10	1.65	2.20	2.65	.0037	adjective	1.58	12.25	-	-	-	-	-
76	dashboard	9	3.06	2.33	2.65	.0038	noun	4.61	9.21	5.25	3.15	5.32	651.98	1.00
77	concessionary	13	2.78	1.37	3.25	.0064	adjective	2.15	14.43	-	-	-	-	-
78	retort	6	2.40	2.03	1.80	.0103	noun	2.75	11.50	-	-	-	628.15	.87
79	extent	6	4.40	2.34	1.70	.0063	noun	1.44	10.72	5.57	3.68	5.00	573.03	.97

	Word	Length	Zipf	PREV	OLD20	BG	PoS	CNC	AoA	VAL	AROU	DOM	LDT RT	LDT Acc
80	mutual	6	3.72	2.14	1.85	.0038	adjective	2.21	8.90	6.48	3.50	6.45	598.86	.95
81	problematic	11	3.43	2.32	3.15	.0050	adjective	2.11	11.63	2.58	4.80	4.65	-	-
82	shiftless	9	1.30	1.62	2.40	.0047	adjective	2.27	12.12	-	-	-	693.12	.70
83	pleasantness	12	1.47	1.59	3.55	.0072	noun	2.00	8.44	-	-	-	-	-
84	nonpayment	10	1.17	1.71	3.60	.0064	noun	2.83	10.00	-	-	-	-	-
85	context	7	4.28	2.24	1.85	.0068	noun	2.17	10.00	5.00	3.18	5.60	597.95	.98
86	shifting	8	3.73	2.34	1.65	.0088	verb	2.86	-	-	-	-	605.50	1.00
87	creamer	7	2.65	1.92	1.45	.0101	noun	4.66	8.72	5.47	2.81	6.09	738.38	.88
88	felicity	8	3.44	1.49	2.10	.0052	name	1.56	-	-	-	-	-	-
89	deferred	8	2.97	2.05	1.75	.0080	verb	2.00	-	-	-	-	666.58	.95
90	gyroscope	9	2.19	1.67	2.75	.0028	noun	4.25	12.69	-	-	-	-	-
91	recalculate	11	1.81	2.15	2.95	.0064	verb	2.93	11.53	-	-	-	-	-
92	frosty	6	3.51	2.35	1.80	.0046	adjective	3.90	6.33	6.15	4.61	5.00	607.38	.98
93	cohesiveness	12	1.60	1.85	3.85	.0088	noun	2.62	-	-	-	-	-	-
94	meld	4	2.19	1.34	1.00	.0060	verb	2.86	11.63	-	-	-	601.62	.34
95	awfulness	9	2.37	1.67	2.80	.0031	noun	2.20	9.67	-	-	-	-	-
96	rolled	6	4.16	2.25	1.45	.0069	verb	3.64	-	-	-	-	546.38	.97
97	orange	6	4.64	2.26	1.40	.0101	noun	4.66	3.26	6.81	4.04	5.58	519.53	.98
98	easily	6	4.69	2.43	1.75	.0061	adverb	1.80	-	-	-	-	-	-
99	reestablish	11	1.70	1.67	3.40	.0077	verb	2.54	10.33	6.14	4.00	6.18	-	-
100	lacquer	7	3.06	1.56	1.85	.0050	noun	4.28	13.19	4.95	3.30	5.00	699.11	.75

D Statistical Power Analysis

We conducted simulations to identify the number of participants required to reach at least 80% power (an arbitrary but commonly used target for statistical power), if we were to carry out the same experiment a large number of times. To match our hypothesis, the planned analysis for this experiment focused on the Congruency-Predictability interaction. A fixed effect coefficient for the interaction in the expected direction would be evidence for a Congruency-dependent effect of Predictability on the N1 that is consistent with a simple predictive coding account. The expected fixed effects coefficients were calculated assuming an interaction between Predictability and image-word Congruency consisting of a .75 μV reduction in N1 amplitude for the most relative to the least predictable congruent trials, with no difference for incongruent trials. Importantly, while we simulated a pattern of effects in which predictability reduced N1 amplitude for picture-congruent words, but not -incongruent words, the interaction term would capture any pattern of results consistent with our predictive coding hypothesis.

To determine the .75 μV effect size, first we decided to simulate the difference as a proportion of the maximum N1 amplitude, because different EEG systems and setups can result in vastly different voltage measurements. Next, to identify a realistic proportional difference at the maximum level of predictability (100% name agreement) between picture-congruent and picture-incongruent words, we considered the design by Kim and Gilley (2013), which is as close to this design as we could find. In their study, 53 participants were presented with highly predictable target words which were either prediction-congruent or prediction-incongruent. Kim and Gilley observed left-lateralised occipitotemporal electrodes' N1 peaks that were less negative when the word was prediction-congruent (-2.6 μV) than when the word was prediction-incongruent (-3.9 μV), equal to a proportional difference of .33. A less comparable, though still possibly informative, study from Kim and Lai (2012) presented 20 participants with 180 high Cloze probability sentences (with 550 ms SOAs such that overlap of ERPs was minimised). The

last word in each sentence was either a highly predictable word, an orthographically similar pseudoword, an orthographically dissimilar pseudoword, or a consonant string nonword. Here, the N1 (170-205 ms) for a left occipitotemporal electrode was shown to be more negative for nonwords and orthographically dissimilar pseudowords (both around -4 μV) than for the predicted word and an orthographically similar pseudoword (both around -3 μV). This is equal to a proportional difference of .25. We decided that other potentially comparable studies, published at the time of the power analysis, were too different in their experimental design, either because they used manipulations other than biasing predictions for specific word forms (Chen et al., 2013, 2015; Segalowitz & Zheng, 2009; Strijkers et al., 2015; Walsh et al., 2020; Wang & Maurer, 2017) or they presented the target items midway through sentences using an SOA of 300 ms or less resulting in overlapping ERPs (Dambacher et al., 2012; Kretzschmar et al., 2015; Sereno et al., 2019).

Given the lack of relevant data, we decided a proportional difference of .15 was a realistic effect size for the difference between picture-congruent and picture-incongruent trials at the maximum level of predictability. In previous participants recorded on the same EEG system for a separate experiment, we observed a mean peak N1 amplitude of around -5 μV. Assuming a proportional difference of .15, we therefore expected a .75 μV reduction in N1 amplitudes at the highest level of predictability, relative to the lowest level of predictability, in the picture-congruent condition. The values we predicted for the extremities of each independent variable are presented in Table 3.

In each iteration of the simulation, we simulated 200 (100 per congruency condition) trials for each of N subjects with subject-, picture-, and word-specific random intercepts and slopes. The predictability values were taken directly from the generated stimuli. The simulation can be understood through reference to the formula that describes the linear mixed effects model:

Table 3

The coding method and predicted N1 amplitudes for the extremities of each predictor variable. As congruency is deviation-coded and there are an equal number of congruent and incongruent trials, the values for $Cong_{spw}$ are presented as between -.5 and .5, though the actual values are likely to differ slightly after observations fitting exclusion criteria are removed (in both the simulation and actual analysis). $Pred_{spw}$ values are calculated as proportion of agreement normalized between 0 and 1.

Congruency	$Cong_{spw}$	Percentage of modal name agreement (%)	$Pred_{spw}$	Predicted N1 amplitude (μV)
Incongruent	-.5	7	0	-5.00
Incongruent	-.5	100	1	-5.00
Congruent	.5	7	0	-5.00
Congruent	.5	100	1	-4.25

$$y_{spw} = \beta_0 + S_{0s} + P_{0p} + W_{0w} + (\beta_1 + S_{1s} + P_{1p})Cong_{spw} + (\beta_2 + S_{2s})Pred_{spw}$$

$$+(\beta_{12} + S_{12s})Cong_{spw}Pred_{spw} + e_{spw}$$

Table 4 explains each term in this model and presents the values simulated for the power analysis. The simulated values for the fixed effects were calculated based on the predictions and coding scheme, and are also presented in Table 4. The simulated values of subject random intercepts were based on mixed effects models for N1 amplitudes in prior research from the lab (*citation removed for double-blinding*), where subject random effects showed much greater variability between subjects than items. The variance for the distribution residuals was also based on estimates from mixed effects models in these analyses. Due to the coding method of the coefficients, the β terms in the table and equation above can be interpreted as follows:

β_0 reflects the average amplitude at the lowest level of predictability,

β_1 reflects the difference between congruent and incongruent trials at the lowest level of

Table 4

The meaning of each term in the design's linear mixed effects model, and the value simulated for the power analysis. Where simulated variables were drawn from distributions, $\sim N(\mu, \sigma)$ indicates that the respective variable's values were drawn from a normal distribution with mean μ and standard deviation σ.

Term	Meaning	Simulated Value (μV)
y_{spw}	Trial-level N1 amplitudes for subject s, picture p, and word w	
β_0	Grand intercept	$= -5$
S_{0s}	Subject random intercept for subject s	$\sim N(0, 2.5)$
P_{0p}	Picture (image) random intercept for picture p	$\sim N(0, 2.5)$
W_{0w}	Word random intercept for word w	$\sim N(0, 2.5)$
β_1	Fixed effect of congruency	$= 0$
S_{1s}	Subject random slope for congruency for subject s	$\sim N(0, .75)$
P_{1p}	Picture (image) random slope for congruency for picture p	$\sim N(0, .5)$
$Cong_{spw}$	Trial-level congruency values (deviation-coded)	
β_2	Fixed effect of predictability	$= .375$
S_{2s}	Subject random slope for predictability for subject s	$\sim N(0, 1)$
$Pred_{spw}$	Trial-level predictability values	
β_{12}	Fixed effect of congruency-predictability interaction	$= .75$
S_{12s}	Subject random slope for congruency-predictability interaction for subject s	$\sim N(0, 1)$
e_{spw}	Residual random noise	$\sim N(0, 3)$

predictability,

β_2 reflects the overall effect of predictability across congruent and incongruent trials, and

β_{12} reflects the difference between congruent and incongruent trials at the highest level of predictability.

In each simulation, simulated participants were pseudo-randomly assigned to stimulus sets 1 and 2 in equal number, or with randomly allocated counts of $\frac{N}{2} - 0.5$ and $\frac{N}{2} + 0.5$ if the number of simulated participants were odd. N varied from 10 to 100 in steps of 5, with 500 iterations run at each value. Before models were fit to simulated data in each iteration, data exclusion was simulated as a random 10% loss of trials. The first 5% simulated data

loss observed in the stimuli validation due to trials being responded to incorrectly or with response times less than 250 ms or greater than 1500 ms. No lower bound for response time exclusions was applied in the EEG experiment, as the word was visible for 1 second before responses are permitted. As a conservative estimate, however, we expected a similar percentage of data loss to that seen in the validation of the picture word stimuli. The remaining 5% of data loss was simulated because, given the participant exclusion criteria, this is the maximum allowable loss of data due to a combination of technical problems with the EEG system. This conservative estimate can be considered a worst-case scenario in terms of EEG data loss. The possibility of participants being excluded was not simulated, as we opted to simply continue collecting data until we reached the desired number of participants, and excluded participants' data would not be analysed. Covarying random effects were simulated using the R package *faux* (DeBruine, 2020). Linear mixed effects models were fit using the same functions, formula, and optimiser as those used for the analysis of the actual data. In the case of non-convergence, models were re-fit without random correlations before significance testing, as this is the action we would take when modelling the actual data. Likelihood ratio Chi-square model comparisons were conducted between the full model and a version of the model lacking the interaction term, and the resulting p values were recorded from each iteration.

Given that the hypothesis was directional, simulated significance tests were performed using one-tailed comparisons with an alpha level of .05. Running only 500 simulations is likely to give noisy estimates of power when simulating data which can vary in many parameters. Since fitting a much larger number of models would be unfeasible due to the time taken to fit each mixed effects model, the underlying relationship between the number of participants and the design's statistical power was estimated by fitting log-linear binomial generalised linear models (GLMs) to all iterations for one-tailed and two-tailed comparisons. Figure 5 depicts the resulting power curves. The power analysis suggested that a sample size of 68 participants (divisible by four, so as to assign an equal number of

participants to each combination of counterbalanced response and stimulus groups) would be sufficient to reach at least 80% power for detecting the effect of interest in the predicted direction with a one-tailed comparison. Specifically, the model predicted that at this number of participants, assuming the predicted effect exists, we could expect 81.72% power (99% confidence interval = [80.46%, 82.91%]).

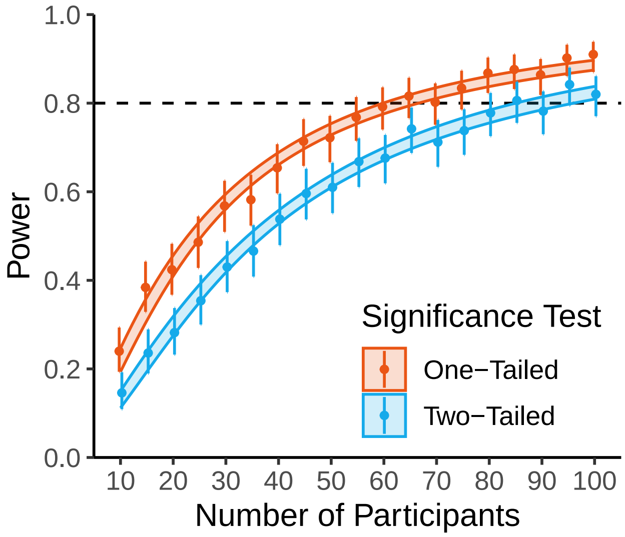

Figure 5

Power curves calculated from the simulations. For comparison, both one-tailed and two-tailed power are presented, though the p *value used in the actual planned analysis is one-tailed. Points (shifted horizontally for visibility) present the observed proportions of simulations which resulted in statistically significant p values. Vertical error bars present 99% binomial confidence intervals of these individual proportions. The coloured lines showing a logarithmic relationship depict the upper and lower bounds of 99% confidence intervals of predicted probabilities from log-linear binomial GLMs fit to the data. The dashed horizontal line highlights the 80% power target.*

We note that due to a lack of relevant data from similar designs, variance-covariance matrices for the power analysis were simulated with all random effects correlations set to zero. To check this did not result in heavily biased estimates, the power analysis was also run with all random effects correlations set to values of .2, .4, .6, and .8. Each of these analyses estimated a strikingly similar relationship between the number of participants and statistical power (Figure 6).

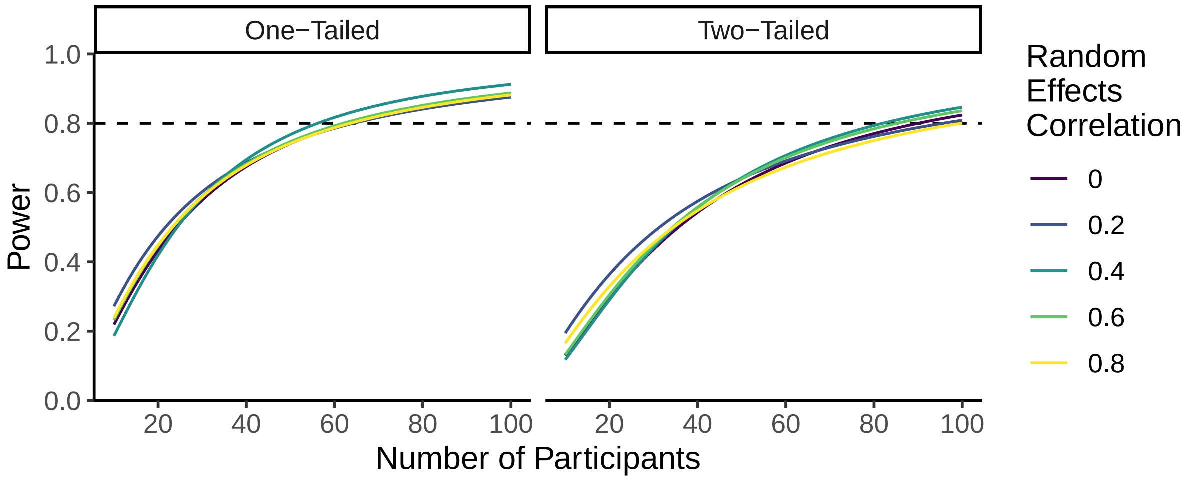

Figure 6

Power curves when all random effect correlations are set to 0, .2, .4, .6, and .8. Each line depicts the predicted relationship between number of participants and power from a single loglinear binomial GLM. As in the original power analysis, results were simulated with N of 10 to 100 in steps of 5, though here with only 100 simulations at each step rather than 500. The overall relationship between the number of participants and the statistical power for finding the predicted interaction remains mostly unchanged across different random effects correlations. As in Figure 5, both one-tailed and two-tailed power are presented, though the p *value used in the experiment is one-tailed. The dashed horizontal line highlights the 80% power target.*

E Instructions given to Participants

Instructions for the localiser and picture-word tasks, shown below, were presented multiple times: at the start of each task, after practice trials, and before the start of each block. The words AFFIRMATIVE and NEGATIVE below were replaced with the text "Left Control" or "Right Control" respectively, depending on which response group the participant was assigned to. In the practice trials, an additional line of text read, "For the practice trials, you will be given feedback on your accuracy for each trial.". For all other trials, this line instead read, "Unlike the practice trials, you will not be given feedback on your accuracy for each trial.".

The instructions for the localiser task were as follows:

In each trial, the following things will happen:
1) You will be shown a picture of a word, nonword, or noise image.
2) The image will turn green.
3) When the image turns green:
Press the AFFIRMATIVE key if the image is of a real word.
OR
Press the NEGATIVE key if it is not of a real word.

Once the image changes colour, try to respond as quickly and accurately as possible.

When you have read these instructions, press the space key to begin...

The instructions for the picture-word task were as follows:

In each trial, the following things will happen:
1) You will be shown a picture of an object for 2 seconds.
2) There will be a short delay.
3) You will be shown a word.
4) The word will turn green.

5) When the word turns green:

Press the AFFIRMATIVE key if the word describes the object you saw.

OR

Press the NEGATIVE key if it does not.

Once the word changes colour, try to respond as quickly and accurately as possible.

When you have read these instructions, press the space key to begin...

F Change to the High-Pass Filter Cut-Off

We originally pre-registered a high-pass filter cut-off of .5 Hz. After pre-registration, we changed this to .1 Hz to address possible artefactual distortions in timings of effects (Rousselet, 2012; Tanner et al., 2015; VanRullen, 2011). Here, we report what our results would have been had we not made this alteration. All other elements of the analysis pipeline match those reported in the manuscript. This analysis reproduced the main finding of an interaction term in the opposite direction to that we expected under a simple predictive coding hypothesis. This suggests that our change to the high-pass filter cut-off did not alter our main results or conclusions.

Maximal Electrode Analysis

The fixed-effect relationship estimated after preprocessing with a high-pass cut-off of .5 Hz is presented in Figure 7, showing a pattern of results similar to that observed with a cut-off of .1 Hz. The model intercept was estimated to be β=-2.83 (*SE*=.46). The fixed effect of Congruency was estimated as β=-.4 (*SE*=.3), and the main effect of Predictability was estimated as β=.3 (*SE*=.26). Importantly, the effect of interest, the interaction between Congruency and Predictability, was in the opposite direction from that hypothesised, estimated as β=-1.24 (*SE*=.46), with a larger effect than that reported in the analysis using a .1 Hz filter (i.e., β=-.79 µV, *SE*=.52).

We also re-fit the Bayesian linear mixed-effects model, as described in the manuscript, to the maximal electrode data extracted after filtering with a .5 Hz cut-off. This revealed a similar posterior distribution to that reported in the manuscript, but with even less of the posterior distribution consistent with the simple predictive coding hypothesis (Figure 8). The median posterior estimate for the Congruency-Predictability interaction was β=-1.23 µV (89% highest density interval = [-1.94, –.48]). We calculated, given this posterior distribution, that the Congruency-Predictability interaction is 239.38 times more likely to be less than 0, than it is to be greater than zero (that is, BF_{01}).

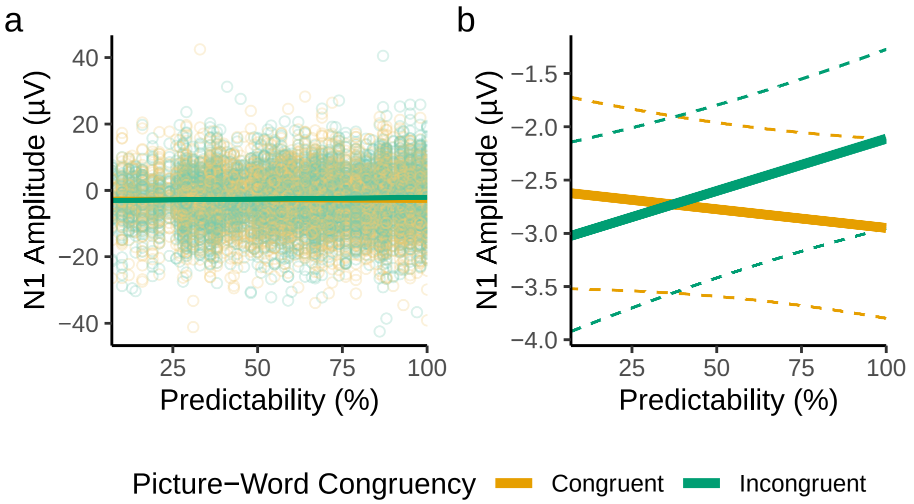

Figure 7

*Maximal electrode results with a .5 Hz cut-off. (**a**) Model-derived fixed-effect predictions, visualised over results from all trials (individual points). (**b**) Fixed-effect predictions visualised alone for visibility. Dashed lines depict the bounds of 95% bootstrapped prediction intervals (5,000 bootstrap samples). For feasibility, bootstrapped predictions were generated from a version of the model that lacked random slopes.*

Full ERP Analysis

We also re-fit the models estimating the full time-course of effects in the region of interest. Importantly, as shown in Figure 9, the Congruency-Predictability interaction term remained negative throughout the N1 period.

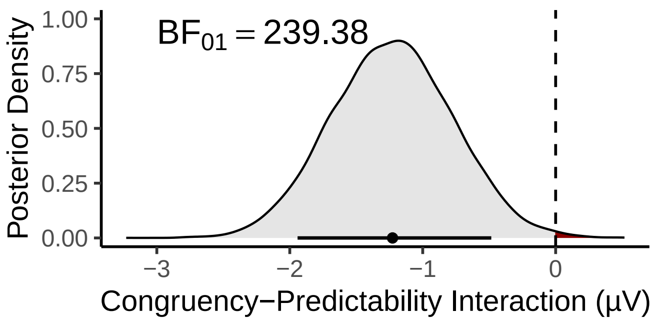

Figure 8

Posterior density for the Congruency-Predictability interaction after filtering with a .5 Hz high-pass cut-off. The point below the density plot depicts the median estimate; the horizontal line shows the 89% HDI of the posterior distribution.

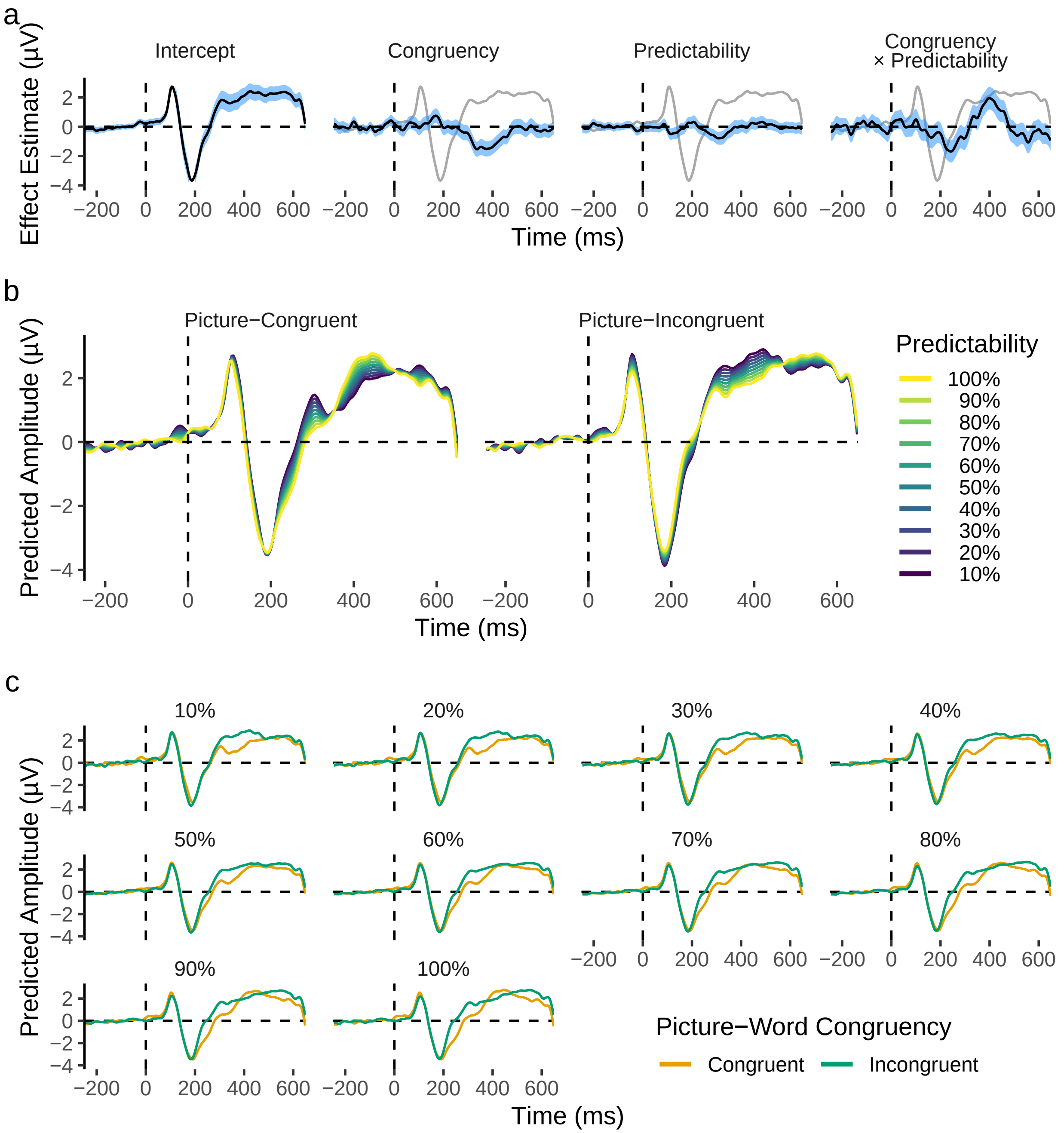

Figure 9

*Time-course of fixed effects from the sample-level analysis of the left-lateralised occipitotemporal region of interest, when filtering with a .5 Hz high-pass cut-off. (**a**) Time-course of fixed-effect estimates. (**b**) Fixed effect predictions showing how predictability affects amplitudes. (**c**) Fixed effect predictions contrasting congruent and incongruent ERPs at each level of predictability.*

G Behavioural Results from the Picture-Word Task

We conducted exploratory behavioural analyses of data from the picture-word EEG task.

Response Times Results

We analysed response times (RTs) to examine whether the pattern of effects was similar to that observed for the behavioural validation experiment (Supplementary Materials B). We fit a Bayesian distributional shifted log-normal model, estimating the same model formula as that described for the behavioural validation experiment for all shifted log-normal parameters (μ, σ, and δ):

```
rt ~ 1 + congruency * predictability +
        (1 + congruency * predictability | subject_id) +
        (1 + congruency | image_id) +
        (1 | word_id),
sigma ~ 1 + congruency * predictability +
        (1 + congruency * predictability | subject_id) +
        (1 + congruency | image_id) +
        (1 | word_id),
ndt ~ 1 + congruency * predictability +
        (1 + congruency * predictability | subject_id) +
        (1 + congruency | image_id) +
        (1 | word_id)
```

The parameter of μ was modelled with an identity link function, while σ and δ were modelled with log link functions. We specified prior distributions based on the posterior distributions from the behavioural validation experiment. Priors for the behavioural analysis of the EEG experiment were not exact replicas of the validation experiment's posteriors, but were rather specified with greater uncertainty than that observed in the validation experiment's posteriors. We decided to specify this uncertainty because of key differences in the task demands: participants in the validation experiment could respond to stimuli without lower limit, whereas in the EEG experiment, responses were only permitted 500 ms after stimulus presentation. As a result of the additional time participants had to consider their responses, and because RTs were measured from the time point at which the

stimulus changed colour, we reasoned that (1) responses would be faster overall in the EEG experiment (reflected in a reduced prior for the δ parameter intercept), and (2) effects observed in the validation experiment would be likely smaller in the EEG experiment. Specifically, fixed and random effect prior distributions for the μ and σ parameters, and random effect priors for δ, were specified such that they were centred on the median estimate from the stimulus validation analysis, but with variance of the random effects ten times that observed in the stimulus validation posterior distributions. The fixed effect prior distributions for the δ parameter were specified to be more uninformative than this, as we expected this parameter to change the most. The prior distribution for the δ intercept was drawn from $\sim N(0, 7.5)$, while the fixed effect slopes' priors also had *SD*s of 7.5, but were centred on the posterior estimates from the stimulus validation analysis. Priors for all correlations of effects were kept as the *brms* default of a flat distribution between -1 and 1. The model was fit with 5 Markov chains, each with 10,000 (7,500 warm-up and 2,500 sampling) iterations. The *adapt_delta* parameter was set to .99, and the *max_treedepth* parameter was set to 10. Summaries of the fixed effect posterior distributions, relative to those of the priors, are shown in Figure 10. Similar results are shown for all random effects in Figure 11.

Results revealed that, although the effects were smaller than in the validation experiment, the main finding was replicated, with low predictability eliciting later RTs for picture-congruent words, to a greater extent than it does for picture-incongruent words (Figure 12a). RTs from the EEG experiment also replicated the difference in spread between picture-congruent and -incongruent RTs at low levels of predictability, with the congruency conditions showing more similar spread in RTs as predictability increases (Figure 12b). Again, this effect was smaller for RTs in the EEG experiment than it was for RTs in the validation experiment. Conversely, the difference in shift observed between picture-congruent and -incongruent words at high predictability in the validation experiment was not observed in the EEG experiment.

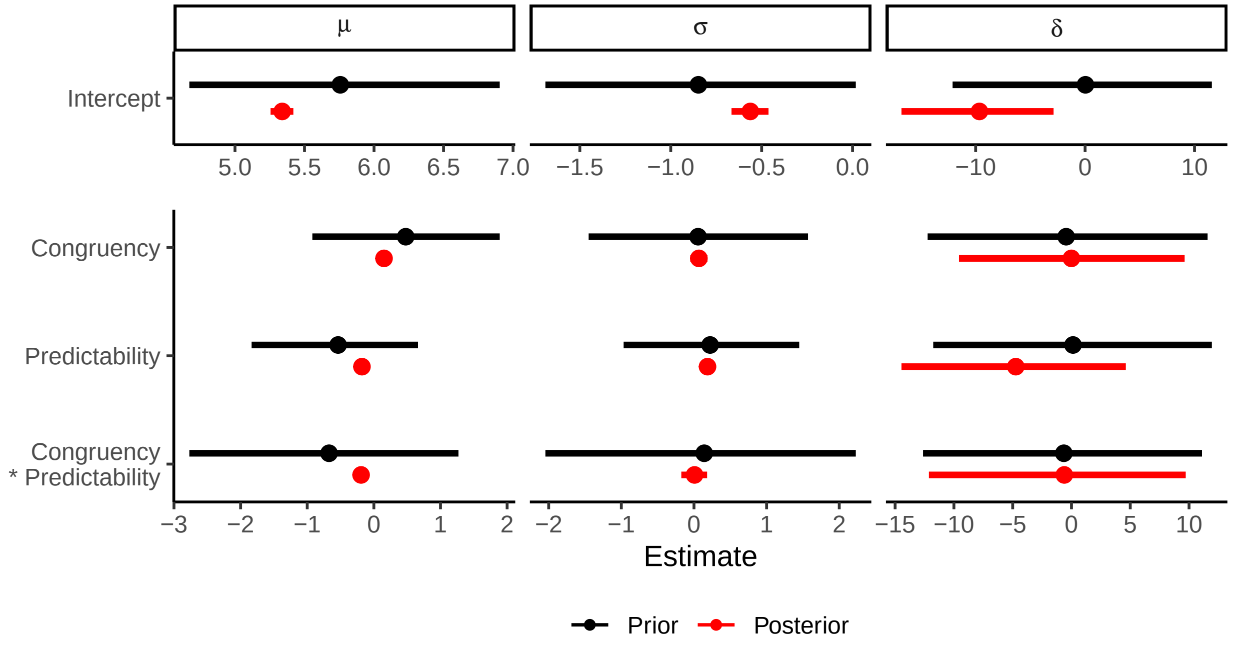

Figure 10

Prior and posterior distributions for all fixed effects estimated in the Bayesian shifted log-normal model of the RT data from the EEG experiment's picture-word task, for the μ*,* σ*, and* δ *parameters. Points depict median estimates, while whiskers depict 89% HDIs, for prior (*black*) and posterior (*red*) distributions.*

Accuracies Results

We similarly analysed accuracies in the picture-word task. We fit a logit-link binomial Bayesian generalised linear mixed effects model (GLMM) to accuracy data, using the same maximal mixed effects formula as that described for the planned analysis of EEG data. All fixed effect prior distributions were specified to be flat, with the exception of the model intercept. As we expected overall accuracy to be very high, we specified the prior distribution for the fixed effect intercept as $\sim N(4, 1)$, where logit 4 would be equivalent to an average accuracy of .982. Priors for the *SD*s of random effects distributions were drawn from Student's t distributions with 3 degrees of freedom, μ of 0, and σ of 2.5. Prior distributions for all correlations were flat (between -1 and 1). The model was fit via *brms*, with 5 chains each sampling for 10,000 iterations (5,000 warmup). The *adapt_delta* parameter was set to .9, and the maximum tree depth (*max_tree_depth*) was set to 10.

Results revealed a main effect of predictability with higher accuracy at higher levels of predictability (Figure 13). An interaction with congruency was also observed, where predictability had a larger effect for picture-congruent than for picture-incongruent words, while accuracy remained more consistent across predictability for picture-incongruent words.

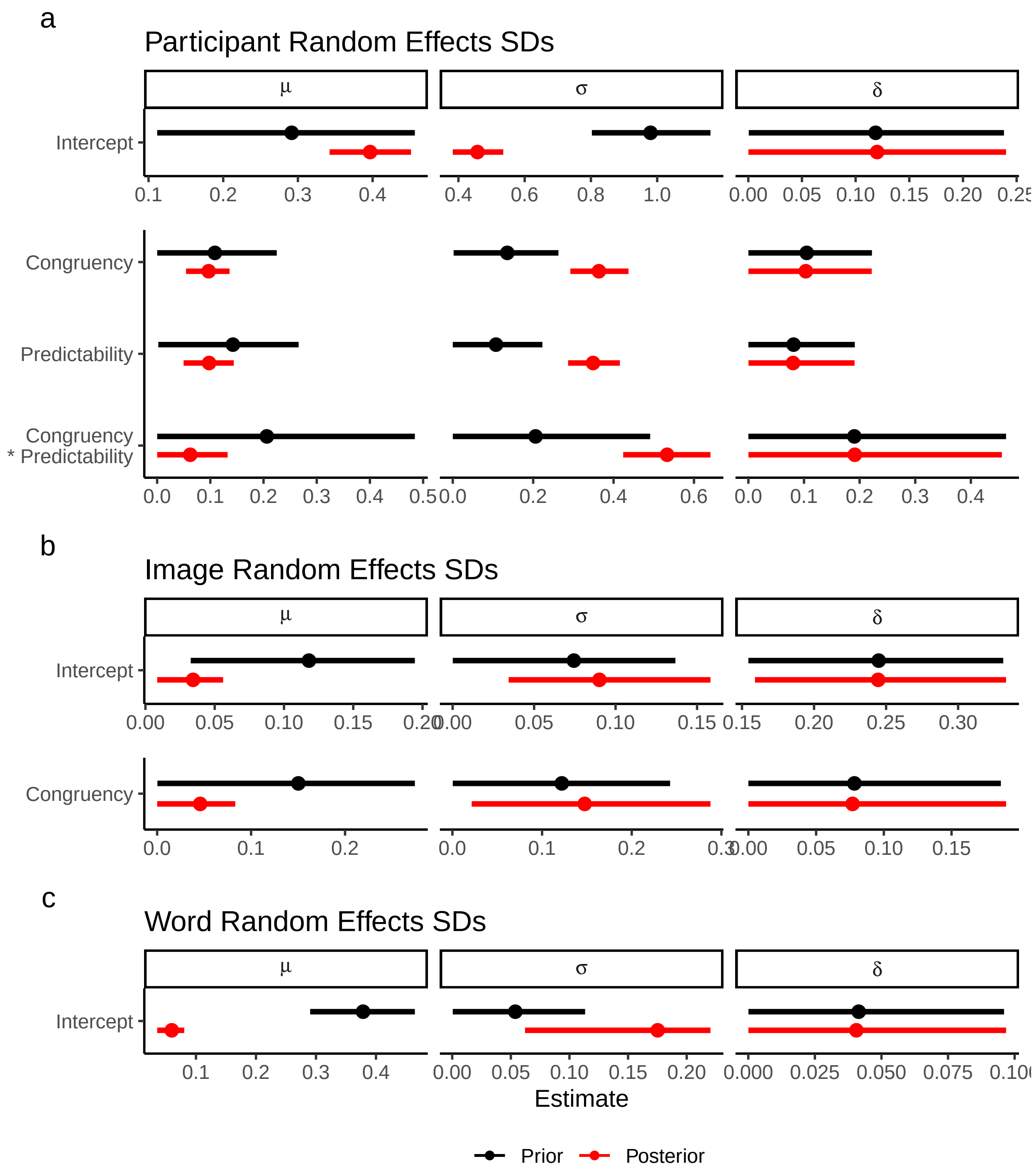

Figure 11

*Prior and posterior distributions for all random effects estimated in the Bayesian shifted log-normal model fit to describe RT data from the EEG experiment's picture-word task, for the μ, σ, and δ parameters. Results are separately for (**a**) participant, (**b**) image, and (**c**) word random effects. Points depict median estimates, while whiskers depict 89% HDIs, for prior* (black) *and posterior* (red) *distributions.*

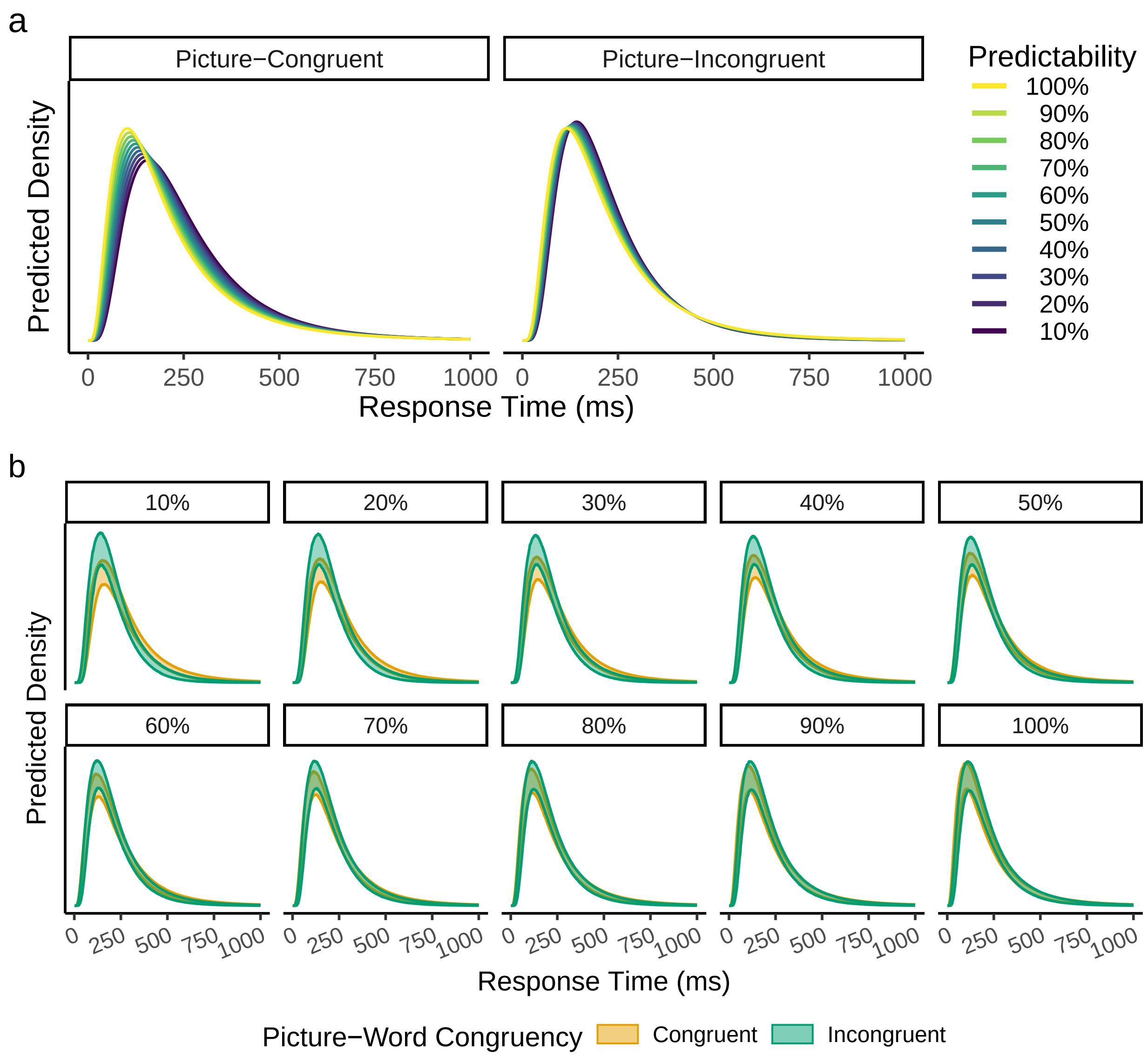

Figure 12

Fixed effect predictions of RT distributions in the EEG experiment. Figure layout is identical to that described for the validation experiment RTs, except that the axis limits for RTs are here limited to ≤1,000 ms. Unlike the validation experiment, where RTs reflect latency from stimulus presentation, RTs here reflect latency from a colour change in the stimulus, that occurred 500 ms after stimulus presentation.

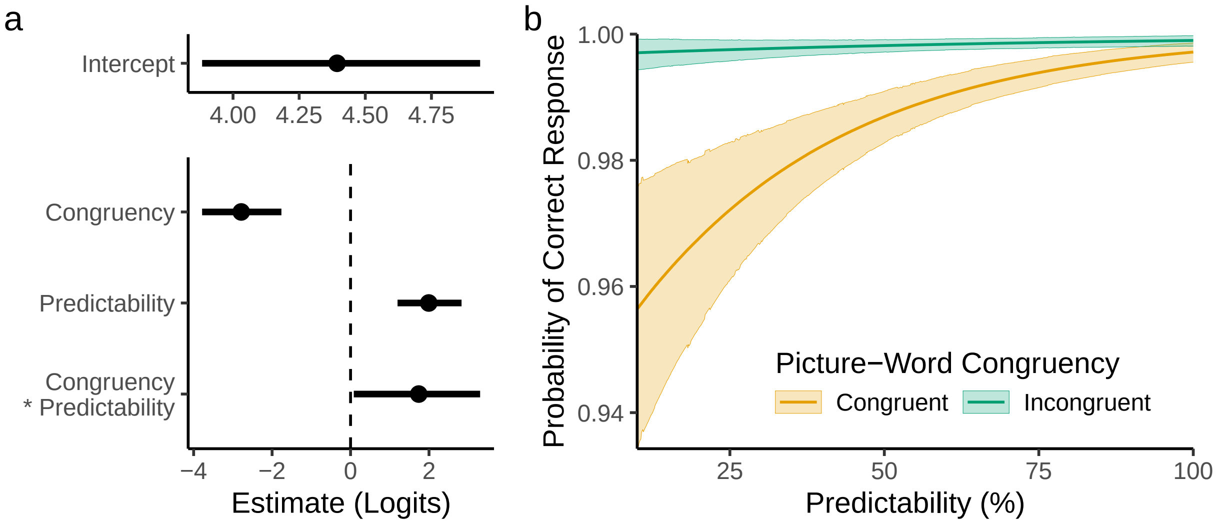

Figure 13

*Fixed effect results for the analysis of accuracies in the picture-word task during the EEG experiment. (**a**) Fixed effect logit estimates, where points depict median estimates and whiskers depict 89% HDIs. (**b**) Model-predicted accuracies, for all levels of predictability in each congruency condition, where the central lines depict median estimates, while the shaded areas depict 89% HDIs.*

H Results from the Localiser Task

We analysed results from localiser task, examining the full time-course of stimulus effects on ERP amplitudes, and patterns of RTs and accuracies.

ERP Results

We analysed the full time-course of stimulus effects in the localiser task, for right- and left-hemispheric occipitotemporal regions of interest. Specifically, separate linear mixed effects models to each time point (256 Hz) via *lme4*, estimating models with the following formula:

```
amplitude ~ 1 + (false_font + noise) * hemisphere +
  (1 | participant_id) +
  (1 | participant_id:electrode_id) +
  (1 | match_set) +
  (1 | item_id)
```

Here, *false_font* and *noise* were deviation-coded variables, comparing the two nonword conditions to the null condition of words (i.e., BACS-font nonwords, and phase-shuffled words, respectively). In this way, the fixed effect slopes represented the difference between words and each non-lexical stimulus type. The deviation-coded variable, *hemisphere*, distinguished observations in the left (*hemisphere*=-.5) and right (*hemisphere*=.5) hemisphere. The *match_set* variable uniquely identified each triplet of matched items. As in the sample-level analysis of the picture-word task, random intercepts were also estimated for each combination of participant and electrode (*participant_id:electrode_id*), and random slopes were excluded for feasibility.

Results revealed that differences between words and phase-shuffled words emerged clearly in the P1 component, with more positive-going amplitudes observed for phase-shuffled words (Figure 14). Differences between words and false-font nonwords, meanwhile, remained small until later, in the N1. In both hemispheres, N1 components were more negative-going for false-font stimuli than for phase-shuffled words. Both positive-going and negative-going

ERP components elicited by words were overall more positive in amplitude for the right hemispheric occipitotemporal electrodes, that is, the P1 was more positive-going, and the N1 less negative-going, in the right hemisphere. The N1 elicited by word stimuli was left-lateralised. An interesting stimulus-hemisphere interaction was observed, wherein ERPs elicited by words showed N1 peak amplitudes most similar to false-font stimuli in the left hemisphere, but most similar to phase-shuffled words in the right-hemisphere. Similar differences in timing were observed in the N1 peak for stimuli across both hemispheres, with phase-shuffled words peaking first, followed by false-font stimuli, and then words. Stimulus effects at occipitotemporal electrodes after the N1 were more consistent across hemispheres, with phase-shuffled words showing the most positive amplitudes, followed by false-font stimuli, which in turn elicited more positive amplitudes than words did, although the difference between words and phase-shuffled words was larger, post-N1, in the right hemisphere. The post-N1 difference between words and false-font nonwords, meanwhile, did not interact with hemisphere except for a brief period around 250 ms.

Behavioural Results

We also analysed stimulus effects on lexical decision RTs and accuracies. Specifically, we fit a logit-link binomial model to trial-level accuracies, and a distributional shifted log-normal model to RTs, with maximal random effects structures.

Accuracies were modelled via a logit-link binomial model (Figure 15a) with an informative prior for the model's logit intercept of $\sim N(5, 1)$ (centred on average accuracy of .993), reflecting the expectation that accuracy overall would be very high. Weakly informative priors were defined for fixed effect slopes ($\sim N(0, 5)$) and for the *SD*s of random effect distributions ($\sim t(5, 0, 1)$). Prior distributions for correlations within the model were specified to be flat. The model was fit with 5 chains, each with 10,000 iterations (7,500 warmup, 2,500 sampling). The *adapt_delta* parameter was set to .99, and the *max_tree_depth* was set to 10. In *brms* syntax, the model estimated coefficients from the following formula:

```
correct ~ 1 + false_font + noise +
  (1 + false_font + noise | participant_id) +
  (1 + false_font + noise | match_set) +
  (1 | item_id)
```

RT data were modelled with a shifted log-normal model (Figure 15b). The parameter of μ was modelled with an identity link function, while σ and δ were modelled with log link functions. The maximal random effects structure was estimated for the distributional parameters μ and σ, whereas the δ parameter was modelled with a global intercept only. This decision was based on persistent divergent transitions in the Hamiltonian Monte Carlo sampler used to explore the model's parameter space. These divergences were caused by the extremely low shift (non-decision time) in the RT data from the EEG experiment, which approached 0 ($-\infty$ on a log scale). This problem is also the likely cause of the high uncertainty for the δ intercept, and also for the parameter coefficients in the analysis of the RT data from the picture-word task. Priors for fixed-effect intercepts were specified to be centred on posterior averages from the picture-word study RT analysis, though with additional uncertainty specified in the distributions to reflect the expectation that RT distributions would differ somewhat from the picture-word task. Specifically, the intercept for μ was specified as $\sim N(5.3, 1)$, σ as $\sim N(-.56, 1)$, and δ as $\sim N(-9, 5)$. Priors for fixed effect slopes were specified as $\sim N(0, 1)$. Prior distributions for the *SD*s of random effects were drawn from Student's t distributions centred on 0, with 5 degrees of freedom and a σ parameter of 1. Prior distributions for all correlations were flat. As with the model of accuracies, the RT model was fit with 5 chains, each with 10,000 iterations (7,500 warmup, 2,500 sampling). The *adapt_delta* parameter was set to .9, and the *max_tree_depth* was set to 10. In *brms* syntax, the model estimated coefficients from the following formula:

```
rt ~ 1 + false_font + noise +
  (1 + false_font + noise | participant_id) +
  (1 + false_font + noise | match_set) +
  (1 | item_id),
sigma ~ 1 + false_font + noise +
```

```
  (1 + false_font + noise | participant_id) +
  (1 + false_font + noise | match_set) +
  (1 | item_id),
ndt ~ 1
```

Results revealed that responses were fastest and most accurate for phase-shuffled words (Figure 16). Responses were slowest and least accurate for word stimuli. RT distributions were similar for false-font and phase-shuffled words, though accuracies for false-font stimuli were closer to those observed for words. Behavioural results overall suggest that participants found it easy to reject phase-shuffled words in lexical decision, but found it relatively more difficult to reject false-font stimuli.

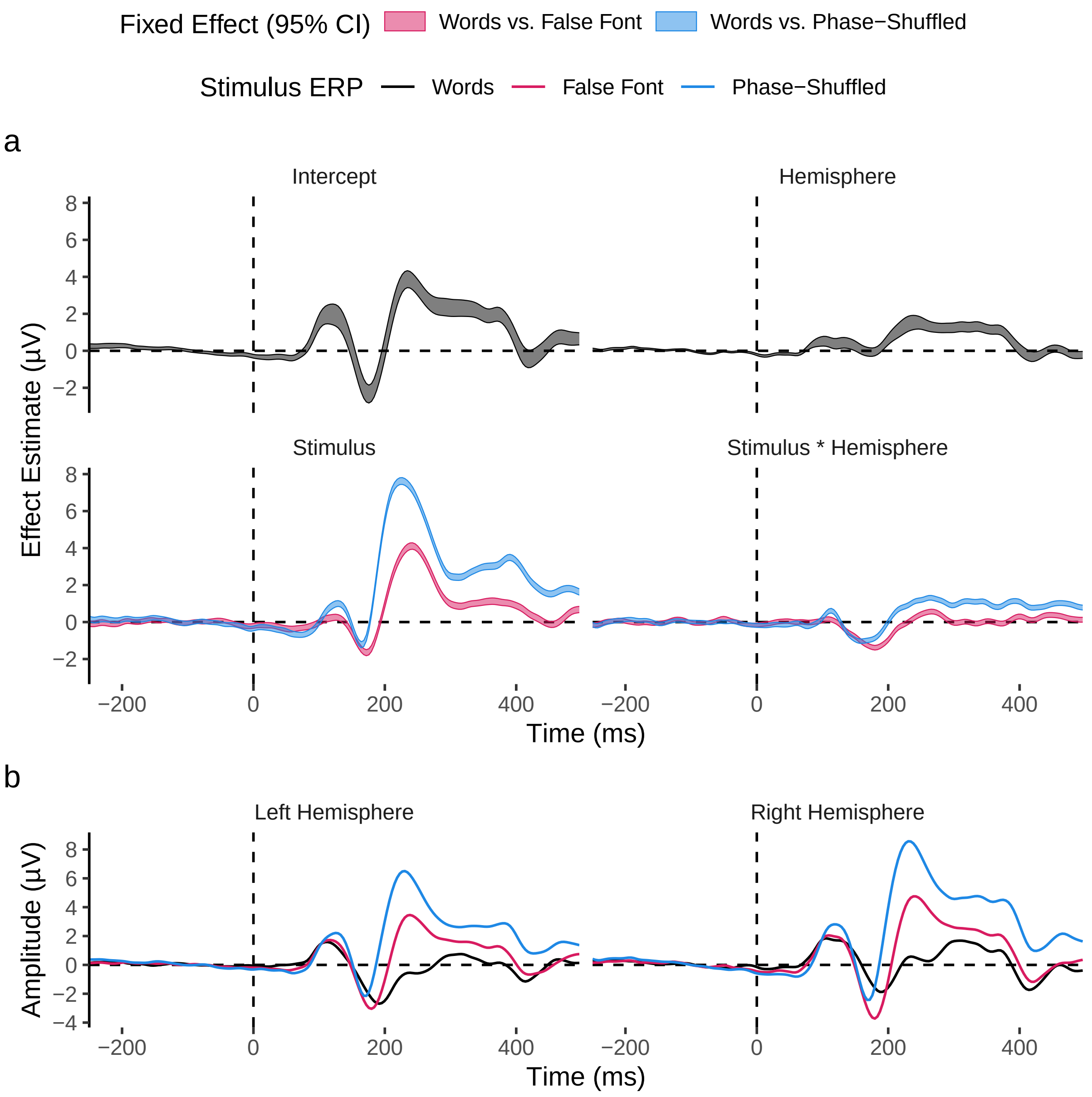

Figure 14

Fixed effect results for ERPs in the localiser task. (a) Fixed effects estimates for each time point, with the shaded areas depicting 95% confidence intervals. (b) Model-derived predictions for ERPs of left- and right-hemispheric occipitotemporal electrodes.

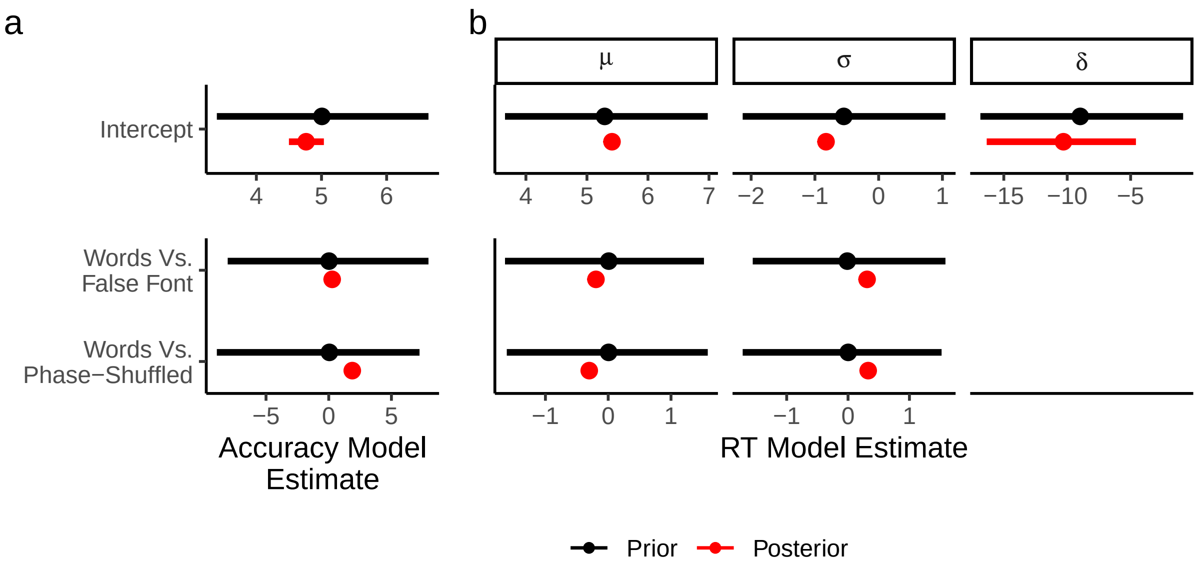

Figure 15

*Prior and posterior distributions for all fixed effects estimated by the (**a**) logit-link Binomial model to describe accuracies and (**b**) Bayesian shifted log-normal model fit to describe RT data from the localiser task. Estimates in (**a**) are in logit units. Estimates in (**b**) are depicted for each shifted log-normal parameter separately. In both panels, points depict median estimates, while whiskers depict 89% HDIs, for prior (*black*) and posterior (*red*) distributions.*

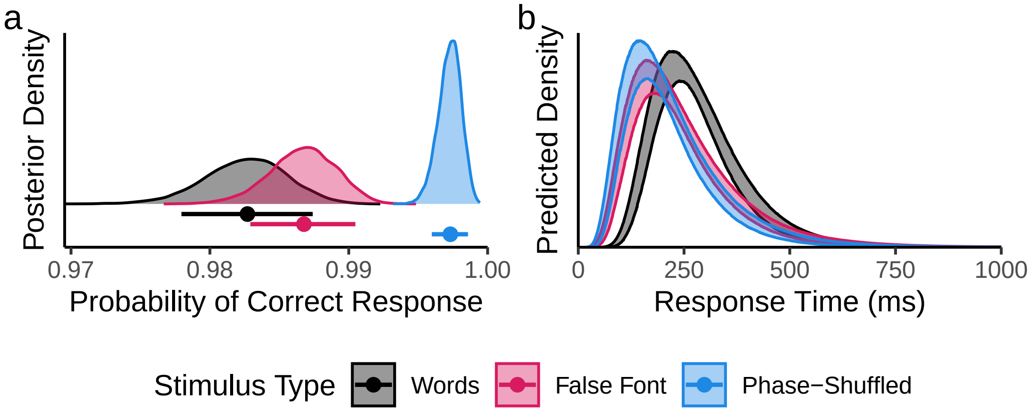

Figure 16

*Fixed effect predictions for behavioural outcomes in the localiser task. (**a**) Posterior distributions for accuracies in the localiser task, where points below densities depict median posterior estimates, while whiskers depict 89% HDIs of posterior samples. (**b**) Predicted RT distributions, where the shaded regions depict 89% HDIs of posterior samples (density values on the* y*-axis begin at 0).*

I Exploratory Alterations to the Maximal Electrode Method

In our planned analysis of the Picture-Word task, we modelled amplitudes recorded at electrodes which also showed, for a given participant, the maximum sensitivity to the word-false-font difference in the Localiser task. We conducted exploratory analyses to examine the impact on our results of different methodologies for extracting trial-level amplitudes from the Picture-Word EEG recordings. For both methodologies, all other parts of the analysis pipeline match those used in the planned analysis. Both methodologies revealed similar results to our planned analysis, with an interaction term in the opposite direction to that expected by our simple predictive coding hypothesis.

Using the Word-Noise Difference

First, rather than identifying maximal electrodes as those that showed maximal sensitivity to the word-false-font difference, we instead identified electrodes that showed maximal sensitivity to the difference between words and phase-shuffled (noise) images. As in the planned analysis, we also used the per-participant peak in this difference to identify the time point at which amplitudes should be extracted. This revealed a similar pattern of effects to that obtained in our planned analysis, with higher predictability eliciting smaller N1s for picture-incongruent words, and larger N1s for picture-congruent words (Figure 17).

To summarise the fixed effects, the model intercept was estimated to be β=-4.1 µV (SE=.55). The interaction was estimated to be β=-1.76 µV (SE=.55). The main effect of congruency was estimated to be β=.75 µV (SE=.37), and the main effect of predictability was β=-.02 µV (SE=.35).

In a Bayesian model identical to that described in the manuscript, but with amplitudes from the alternative maximal electrodes as the outcome variable, the Posterior distribution for the Predictability-Congruency was estimated to be β=-1.73 µV (89% highest density interval = [-2.6, -.88]; 1562 times more likely to be less than 0 than it is to be greater than zero **(Figure 18)**.

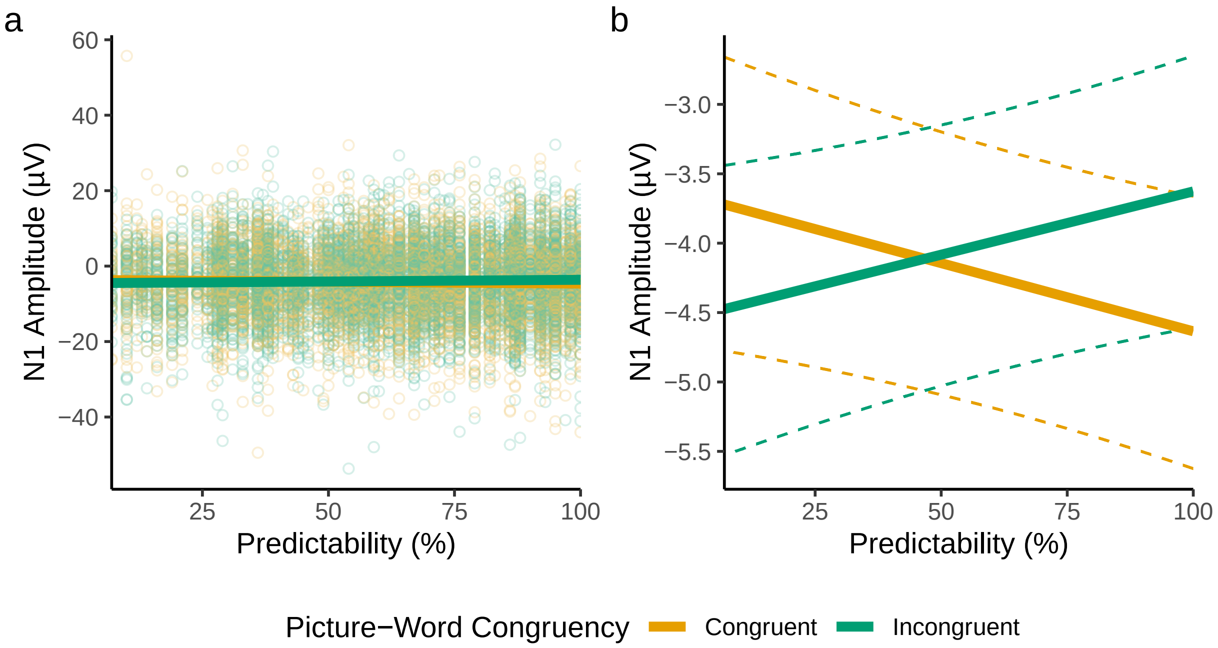

Figure 17

*Results using the word-noise difference to identify maximal electrodes. (**a**) Model-derived fixed-effect predictions, visualised over results from all trials (individual points). (**b**) Fixed-effect predictions visualised alone for visibility. Dashed lines depict the bounds of 95% bootstrapped prediction intervals (5,000 bootstrap samples). For feasibility, bootstrapped predictions were generated from a version of the model without random slopes.*

Using Region-of-Interest Averages

Second, we calculated trial-level amplitudes as the average amplitude across all electrodes in the left-hemispheric occipitotemporal region of interest, across all time points in a 120-200 ms N1 window. Again, this analysis revealed very similar results to our planned analysis **(Figure 19)**.

Here, the model intercept was estimated to be β=-2.95 µV (*SE*=.27). The interaction was estimated to be β=-1.03 µV (*SE*=.3). The main effect of congruency was estimated to be β=-.12 µV (*SE*=.21), and the main effect of predictability was β=ß.05 µV (*SE*=.19).

In a Bayesian model identical to that described in the manuscript, but with the ROI average as the outcome variable, the Posterior distribution for the Predictability-Congruency was estimated to be β=-1.03 µV (89% highest density interval

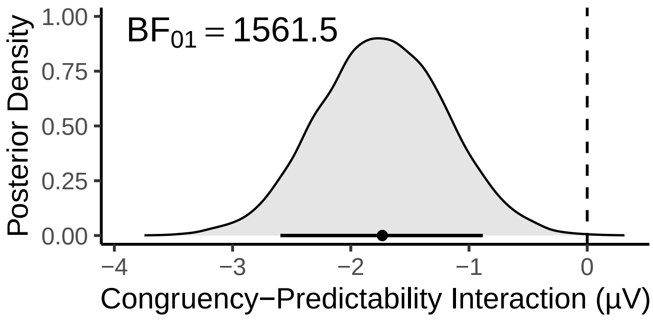

Figure 18

The region of the posterior distribution consistent with the predictive coding hypothesis (where β>0) is highlighted in red. The point and horizontal line below the density plot depict respectively the median estimate and 89% highest density interval of the posterior distribution.

= [-1.52, -.058]; 2082 times more likely to be less than 0 than it is to be greater than zero **(Figure 20)**.

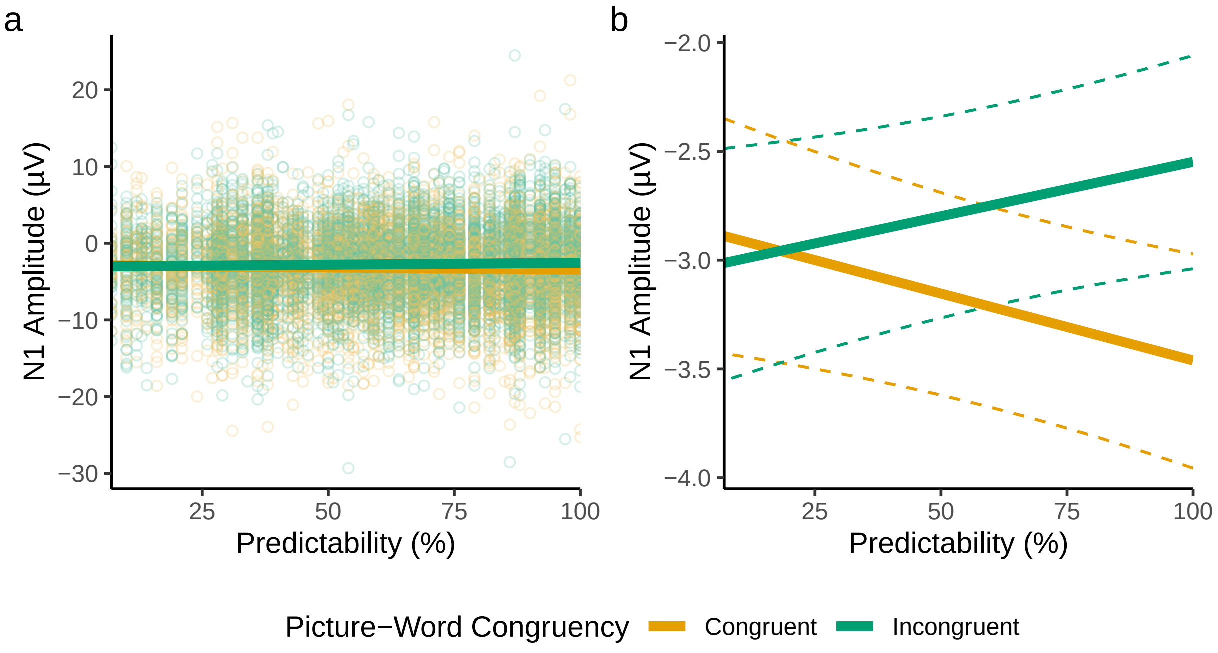

Figure 19

*Results from trial-level models of region-of-interest averages in the N1 window. (**a**) and (**b**) are as described in Figure 17.*

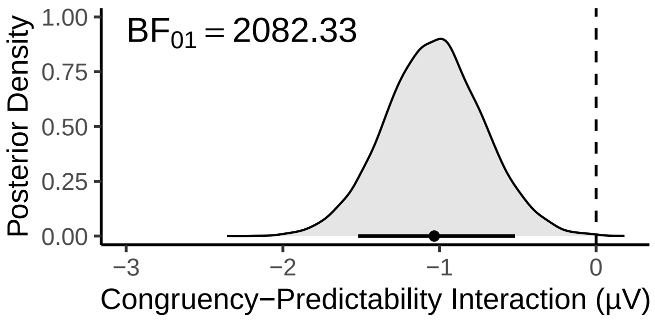

Figure 20

The region of the posterior distribution consistent with the predictive coding hypothesis (where $\beta>0$) is highlighted in red. The point and horizontal line below the density plot depict respectively the median estimate and 89% highest density interval of the posterior distribution.

J Model Estimates for the N400

The exploratory scalp-wide analysis of ERPs revealed a Congruency-Predictability interaction during the period of the N400 component. To more clearly interpret this effect, we generated topographic plots of estimated N400 amplitudes at different levels of predictability, for picture-congruent and -incongruent words separately (**Figure 21**).

Figure 21

Topographic plots of model estimates at 400 ms.

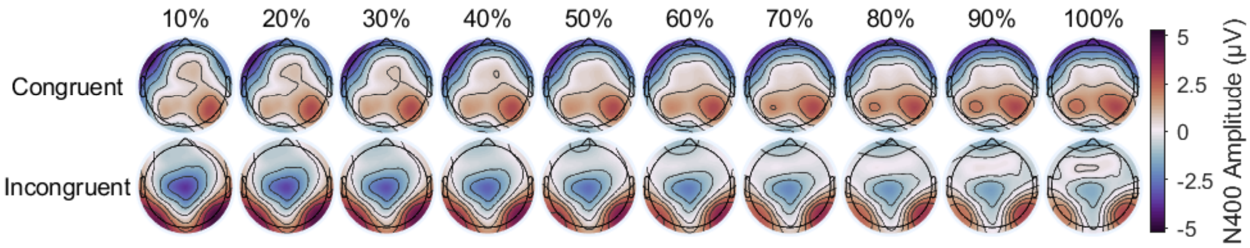

Estimates are shown for picture-congruent and -incongruent words separately, at levels of predictability from 10 to 100%.

Results showed that picture-incongruent words elicited a clear N400 difference from picture-congruent words, and that this difference was largest at low levels of Predictability, and smallest at high levels of Predictability.

K Checking ERPs Time-Locked to Pictures

We conducted an exploratory analysis of the ERPs observed when the evoked signals are time-locked to the onset of pictures that preceded words. The reasoning behind this analysis was twofold. First, we considered that this may reveal an effect of Predictability (Cheng et al., 2010), since name agreement is a feature of the picture stimuli in isolation, as well as of picture-word pairs. Such an effect would have affected the baseline period of the word ERP, and may have influenced estimates of Predictability. Second, we wanted to check that there were no effects of Congruency prior to word presentation. Because we presented words *after* pictures, we reasoned that there should be no effect of Congruency or the Congruency-Predictability interaction in the ERPs elicited by pictures.

To examine picture-elicited ERPs, we fit linear mixed-effects models like those described in the section, *Exploratory Scalp-Wide Analysis*, of the manuscript, but with ERPs time-locked to picture presentation rather than word presentation.

Results (**Figure 23**) revealed an ERP with high positivity over posterior regions. As sometimes seen in ERPs elicited by images (e.g., Cheng et al., 2010; Kaufmann et al., 2009; Rousselet et al., 2004; Weinberg & Hajcak, 2010), the N1 deflection was so small, relative to the P1, that posterior amplitudes remained positive throughout. Consistent with findings reported by Cheng et al. (2010), we found evidence of predictability (i.e., name agreement) effects on the picture ERP. We note, however, that Cheng et al. (2010) found evidence for more positive amplitudes across most of the scalp and time-course. In contrast, we found evidence for more negative amplitudes at occipitotemporal sites, and, conversely, more positive amplitudes in central and frontal locations. By the time of stimulus presentation, the effect of predictability showed a very noisy pattern of differences between midline and peripheral electrodes, similar in magnitude to effects of Congruency and the Congruency-Predictability interaction estimated for the same time point. As expected, we observed no clear effects of Congruency, or of the Congruency-Predictability interaction, on the picture ERP.

Figure 22

Time-course of scalp-wide fixed-effects estimates for picture ERPs.

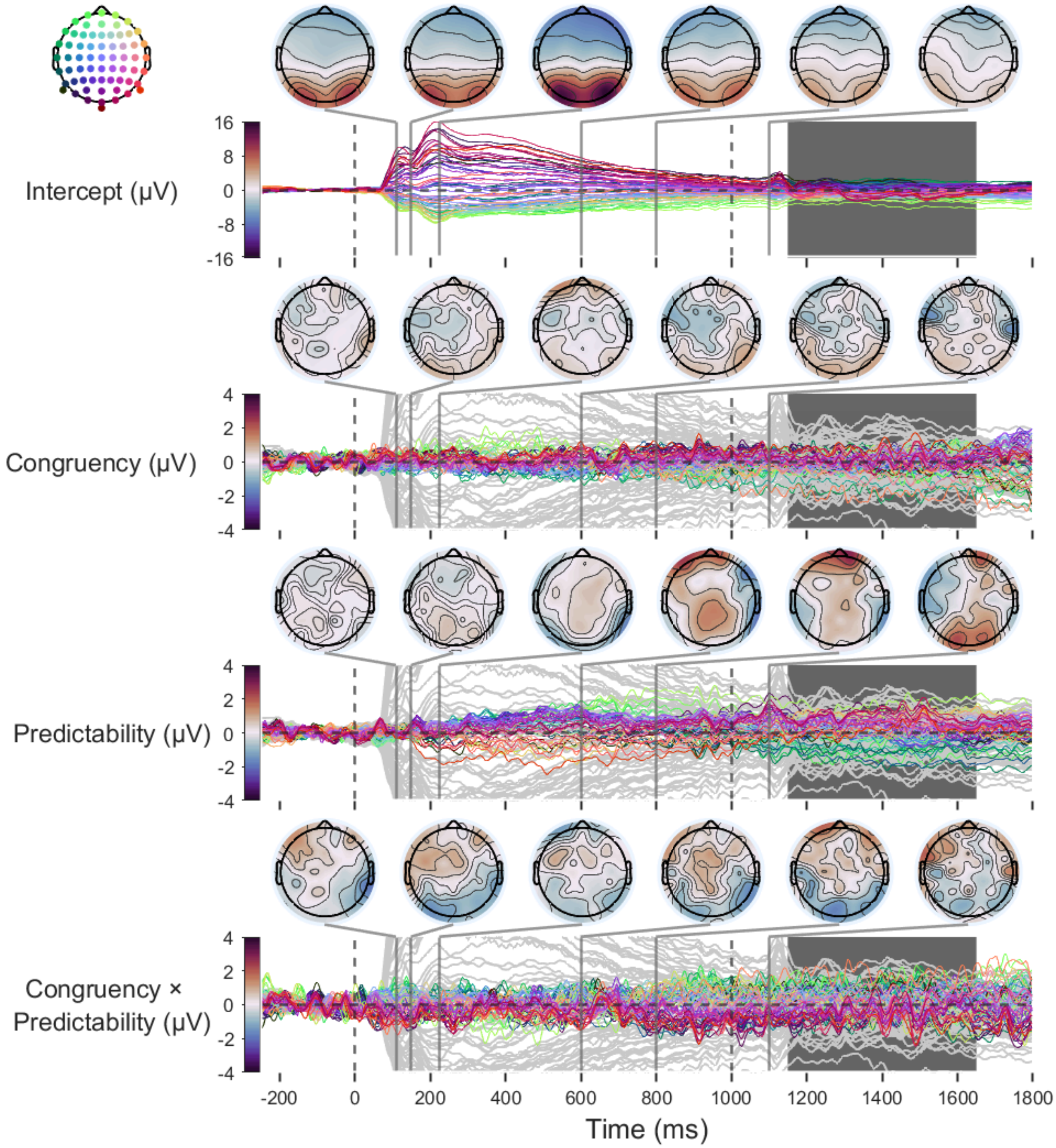

The first dashed vertical line (0 ms) indicates stimulus (picture) onset. The second dashed vertical line (1000 ms) indicates the time-point at which the picture was replaced with a fixation point. The large dark grey period from 1150 to 1650 ms reflects the jittered period during which words were presented. Topographic plots of fixed effects are highlighted at key time-points. Model intercepts (reflecting amplitudes at the lowest level of Predictability) are depicted as grey lines on each panel to provide a reference for timing and magnitude of effects. Note that axis limits for the fixed-effect slopes are reduced by four times for visibility of any small effects.

Although the estimates are noisy, we consider it possible that a sustained effect of Predictability influenced the ERPs elicited by words. Indeed, such an effect may even index preactivation processes that underlie prediction effects on the word N1. Nevertheless, the Congruency-Predictability interaction we used to test our hypotheses can only have emerged after word onset.

L Checking ERPs Time-Locked to Responses

We considered that ERPs elicited by words may have also included signals associated with participants preparing to initiate their response. To examine this we calculated ERPs time-locked to participants' button presses, via linear mixed-effects models like those described in the section, *Exploratory Scalp-Wide Analysis*, of the manuscript. However, as we were interested in ERPs prior to the event, we did not include any baseline correction. Results showed that, beginning around 400 ms prior to response, picture-incongruent words elicited more negative-going centroparietal amplitudes. Given that the colour change, after which participants could respond, occurred at 500 ms, this effect is likely to reflect the large and long-lasting N400 effect of Congruency observed in the word ERPs.

The effect of Predictability, and the Congruency-Predictability interaction, did not show clear effects in the ERP time-locked to responses. Effects of these variables on the word ERP are likely not reflected in the response ERP because, relative to the N400 effect, effects of Predictability and the Congruency-Predictability interaction were smaller and more transient. As a result, these effects are averaged out by variability in response times, which introduces jitter to effects elicited by words.

Figure 23

Time-course of scalp-wide fixed-effects estimates for Response ERPs.

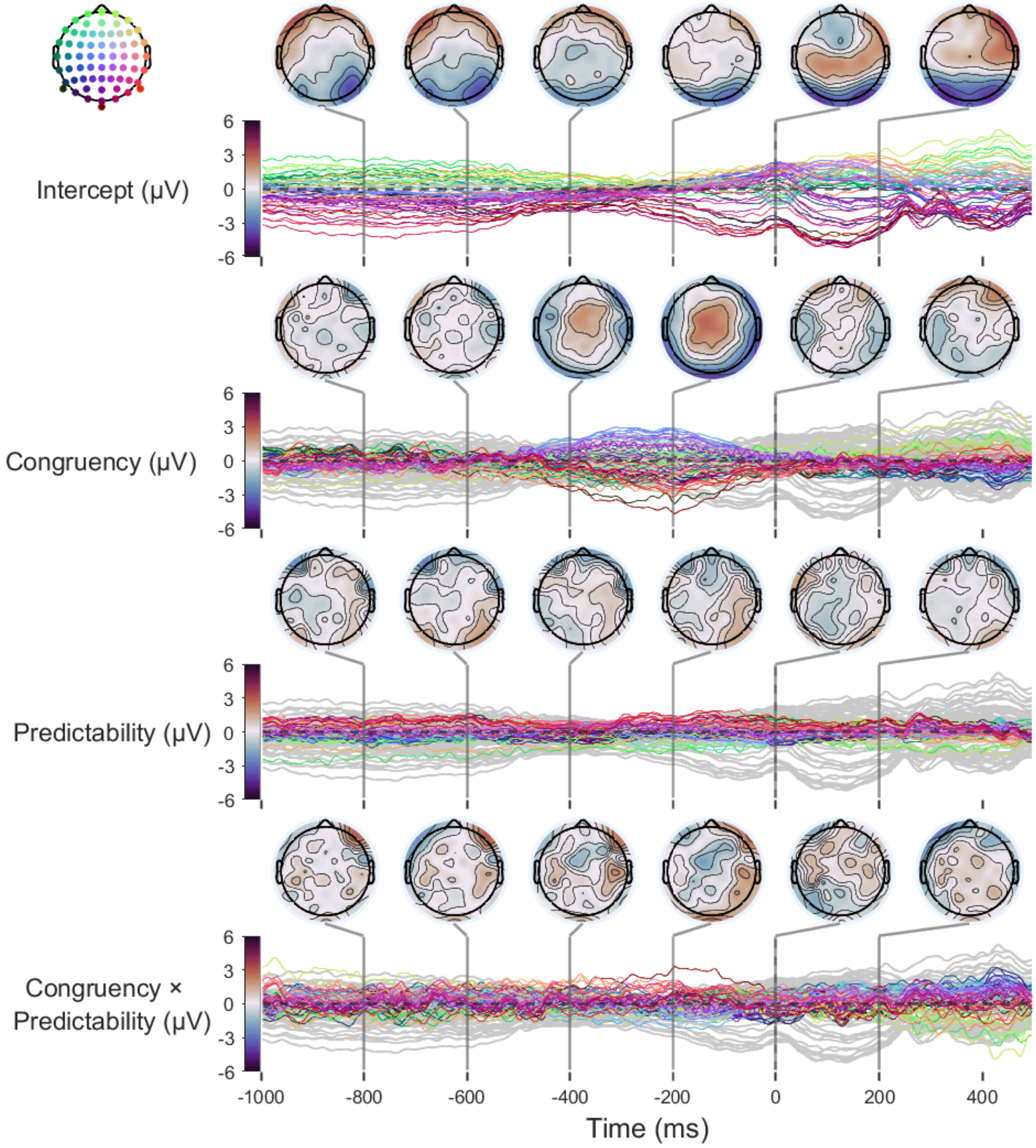

Model intercepts (reflecting amplitudes at the lowest level of Predictability) are depicted as grey lines on each panel to provide a reference for timing and magnitude of effects. Note that axis limits for the fixed-effect slopes are reduced by four times for visibility of any small effects.

References

Brodeur, M. B., Guérard, K., & Bouras, M. (2014). Bank of Standardized Stimuli (BOSS) phase ii: 930 new normative photos. *PLoS ONE*, *9*(9), 1–10. https://doi.org/10.1371/journal.pone.0106953

Brysbaert, M., Mandera, P., McCormick, S. F., & Keuleers, E. (2019). Word prevalence norms for 62,000 English lemmas. *Behavior Research Methods*, *51*(2), 467–479. https://doi.org/10.3758/s13428-018-1077-9

Brysbaert, M., Warriner, A. B., & Kuperman, V. (2014). Concreteness ratings for 40 thousand generally known English word lemmas. *Behavior Research Methods*, *46*(3), 904–911. https://doi.org/10.3758/s13428-013-0403-5

Bürkner, P.-C. (2017). Brms: An R package for Bayesian multilevel models using Stan. *Journal of Statistical Software*, *80*(1), 1–28. https://doi.org/10.18637/jss.v080.i01

Chen, Y., Davis, M. H., Pulvermüller, F., & Hauk, O. (2013). Task modulation of brain responses in visual word recognition as studied using EEG/MEG and fMRI. *Frontiers in Human Neuroscience*, *7*, 1–14. https://doi.org/10.3389/fnhum.2013.00376

Chen, Y., Davis, M. H., Pulvermüller, F., & Hauk, O. (2015). Early visual word processing is flexible: Evidence from spatiotemporal brain dynamics. *Journal of Cognitive Neuroscience*, *27*(9), 1738–1751. https://doi.org/10.1162/jocn_a_00815

Cheng, X., Schafer, G., & Akyürek, E. G. (2010). Name agreement in picture naming: An ERP study. *International Journal of Psychophysiology*, *76*(3), 130–141. https://doi.org/10.1016/j.ijpsycho.2010.03.003

Dambacher, M., Dimigen, O., Braun, M., Wille, K., Jacobs, A. M., & Kliegl, R. (2012). Stimulus onset asynchrony and the timeline of word recognition: Event-related potentials during sentence reading. *Neuropsychologia*, *50*(8), 1852–1870. https://doi.org/10.1016/j.neuropsychologia.2012.04.011

De Deyne, S., Navarro, D. J., Perfors, A., Brysbaert, M., & Storms, G. (2019). Measuring the associative structure of English: The "Small World of Words" norms for word association. *Behavior Resarch Methods*, *51*(3), 987–1006. https://doi.org/10.3758/s13428-018-1115-7

DeBruine, L. (2020). Faux: Simulation for factorial designs. https://doi.org/10.5281/zenodo.2669586

Kaufmann, J. M., Schweinberger, S. R., & Burton, A. M. (2009). N250 ERP correlates of the acquisition of face representations across different images. *Journal of Cognitive Neuroscience*, *21*(4), 625–641. https://doi.org/10.1162/jocn.2009.21080

Keuleers, E., Lacey, P., Rastle, K., & Brysbaert, M. (2012). The British Lexicon Project: Lexical decision data for 28,730 monosyllabic and disyllabic English words. *Behavior Research Methods*, *44*(1), 287–304. https://doi.org/10.3758/s13428-011-0118-4

Kim, A. E., & Gilley, P. M. (2013). Neural mechanisms of rapid sensitivity to syntactic anomaly. *Frontiers in Psychology*, *4*, 1–15. https://doi.org/10.3389/fpsyg.2013.00045

Kim, A. E., & Lai, V. (2012). Rapid interactions between lexical semantic and word form analysis during word recognition in context: Evidence from ERPs. *Journal of Cognitive Neuroscience*, *24*(5), 1104–1112. https://doi.org/10.1162/jocn_a_00148

Kretzschmar, F., Schlesewsky, M., & Staub, A. (2015). Dissociating word frequency and predictability effects in reading: Evidence from coregistration of eye movements and EEG. *Journal of Experimental Psychology : Learning, Memory, and Cognition*, *41*(6), 1648–1662. https://doi.org/10.1037/xlm0000128

Kuperman, V., Stadthagen-Gonzalez, H., & Brysbaert, M. (2012). Age-of-acquisition ratings for 30,000 English words. *Behavior Research Methods*, *44*(4), 978–990. https://doi.org/10.3758/s13428-012-0210-4

Pastore, M., & Calcagnì, A. (2019). Measuring distribution similarities between samples: A distribution-free overlapping index. *Frontiers in Psychology*, *10*(1089), 1–8. https://doi.org/10.3389/fpsyg.2019.01089

Rousselet, G. A. (2012). Does filtering preclude us from studying ERP time-courses? *Frontiers in Psychology*, *3*, 1–9. https://doi.org/10.3389/fpsyg.2012.00131

Rousselet, G. A., Thorpe, S. J., & Fabre-Thorpe, M. (2004). Processing of one, two or four natural scenes in humans: The limits of parallelism. *Vision Research*, *44*(9), 877–894. https://doi.org/10.1016/j.visres.2003.11.014

Segalowitz, S. J., & Zheng, X. (2009). An ERP study of category priming: Evidence of early lexical semantic access. *Biological Psychology*, *80*(1), 122–129. https://doi.org/10.1016/j.biopsycho.2008.04.009

Sereno, S. C., Hand, C. J., Shahid, A., Mackenzie, I. G., & Leuthold, H. (2019). Early EEG correlates of word frequency and contextual predictability in reading. *Language, Cognition and Neuroscience*, *35*(5), 625–640. https://doi.org/10.1080/23273798.2019.1580753

STAN Development Team. (2023). Stan Modeling Language Users Guide and Reference Manual, 2.32. https://mc-stan.org

Strijkers, K., Bertrand, D., & Grainger, J. (2015). Seeing the same words differently: The time course of automaticity and top-down intention in reading. *Journal of Cognitive Neuroscience*, *27*(8), 1542–1551. https://doi.org/10.1162/jocn_a_00797

Tanner, D., Morgan-Short, K., & Luck, S. J. (2015). How inappropriate high-pass filters can produce artifactual effects and incorrect conclusions in ERP studies of language and cognition. *Psychophysiology*, *52*(8), 997–1009. https://doi.org/10.1111/psyp.12437

Taylor, J. E., Beith, A., & Sereno, S. C. (2020). LexOPS: An R package and user interface for the controlled generation of word stimuli. *Behavior Research Methods*, *52*, 2372–2382. https://doi.org/10.3758/s13428-020-01389-1

van Heuven, W. J., Mandera, P., Keuleers, E., & Brysbaert, M. (2014). SUBTLEX-UK: A new and improved word frequency database for British English. *Quarterly Journal of Experimental Psychology*, *67*(6), 1176–1190. https://doi.org/10.1080/17470218.2013.850521

VanRullen, R. (2011). Four common conceptual fallacies in mapping the time course of recognition. *Frontiers in Psychology*, *2*, 1–6. https://doi.org/10.3389/fpsyg.2011.00365

Vidal, C., Content, A., & Chetail, F. (2017). BACS: The Brussels Artificial Character Sets for studies in cognitive psychology and neuroscience. *Behavior Research Methods*, *49*(6), 2093–2112. https://doi.org/10.3758/s13428-016-0844-8

Walsh, K. S., McGovern, D. P., Clark, A., & O'Connell, R. G. (2020). Evaluating the neurophysiological evidence for predictive processing as a model of perception. *Annals of the New York Academy of Sciences*, *1464*(1), 242–268. https://doi.org/10.1111/nyas.14321

Wang, F., & Maurer, U. (2017). Top-down modulation of early print-tuned neural activity in reading. *Neuropsychologia*, *102*, 29–38. https://doi.org/10.1016/j.neuropsychologia.2017.05.028

Warriner, A. B., Kuperman, V., & Brysbaert, M. (2013). Norms of valence, arousal, and dominance for 13,915 English lemmas. *Behavior Research Methods*, *45*(4), 1191–1207. https://doi.org/10.3758/s13428-012-0314-x

Weinberg, A., & Hajcak, G. (2010). Beyond good and evil: The time-course of neural activity elicited by specific picture content. *Emotion (Washington, D.C.)*, *10*(6), 767–782. https://doi.org/10.1037/a0020242

Yarkoni, T., Balota, D., & Yap, M. (2008). Moving beyond Coltheart's N: A new measure of orthographic similarity. *Psychonomic Bulletin and Review*, *15*(5), 971–979. https://doi.org/10.3758/PBR.15.5.971
